# Second-generation lysocins as therapeutics for treating *Pseudomonas aeruginosa* infections

**DOI:** 10.1128/aac.01312-25

**Published:** 2025-11-12

**Authors:** Ryan D. Heselpoth, Chad W. Euler, Vincent A. Fischetti

**Affiliations:** 1 Laboratory of Bacterial Pathogenesis and Immunology, The Rockefeller University 5929, New York, New York, USA; 2 Department of Medical Laboratory Sciences, Hunter College, City University of New York 5924, New York, New York, USA; 3 Department of Microbiology and Immunology, Weill Cornell Medical College 12295 , New York, New York, USA; Entasis, Big Bay, Michigan, USA

**Keywords:** lysocin, lysin, bacteriocin, pyocin, protein engineering, drug delivery, *Pseudomonas aeruginosa*, antimicrobial, cystic fibrosis, antibiotic resistance

## Abstract

*Pseudomonas aeruginosa* is a leading cause of nosocomial infections, including pneumonia and urinary tract infections, and the primary cause of morbidity and mortality in cystic fibrosis patients. The emergence of multidrug-resistant strains makes these infections life-threatening. To overcome this challenge, lysocins can be employed as novel antipseudomonals. Lysocins use components of the pyocin antimicrobial system to deliver bacteriophage lysins to their peptidoglycan substrate in *Pseudomonas*. Peptidoglycan cleavage causes membrane destabilization, cytoplasmic leakage, and disruption of the proton motive force, thereby killing the cell. In our previous proof-of-concept study, the PyS2-GN4 lysocin killed only one-third of *P. aeruginosa* strains due to the targeted receptor. This limitation can now be circumvented by engineering second-generation lysocins that bind and translocate through highly conserved *Pseudomonas*-specific receptors. One lysocin, PyS5-I-GN4, uses a single domain from pyocin S5 to deliver the GN4 lysin through the conserved ferric pyochelin transporter, consequently killing 95% of multidrug-resistant clinical isolates tested. Importantly, PyS5-I-GN4 displayed antibiofilm properties and was bactericidal in serum and lung surfactant. Serum inactivation observed for lysins is not seen for lysocins, making this approach more effective for treating systemic Gram-negative bacterial infections. Despite its broadened pseudomonal strain coverage, PyS5-I-GN4 demonstrated narrow-spectrum antibacterial activity toward *P. aeruginosa* only and lacked cytotoxicity toward human cells. A single dose of lysocin was protective and reduced bacteria multiple log_10_-fold in the lungs and secondary organs in a neutropenic murine lung infection model. These findings support lysocins as therapeutics for *P. aeruginosa* and provide insight into designing future constructs for other Gram-negative pathogens.

## INTRODUCTION

The ubiquitous Gram-negative bacterium *Pseudomonas aeruginosa* is a common nosocomial pathogen that causes a wide array of life-threatening infections, including pneumonia, bloodstream infections, urinary tract infections, burn wound infections, and surgical site infections (1–3). Moreover, *P. aeruginosa* is the leading cause of morbidity and mortality in patients with cystic fibrosis (CF), which is the most frequent lethal hereditary disease in Caucasian populations (4). This bacterial species alone infects more than 50% of CF patients ages 18 years and older in the United States (5). A more rapid decline in pulmonary function observed with CF patients who acquire *P. aeruginosa* infections is associated with a 2.6-fold higher risk of death and a decreased life expectancy of 30 years, compared with 40 years for non-colonized patients (6, 7).

The diminishing antipseudomonal efficacy associated with antibiotics used clinically for treating *P. aeruginosa* infections can be attributed to a combination of intrinsic, acquired, and adaptive resistance mechanisms utilized by the bacteria (8). Antibiotic tolerance and resistance by *P. aeruginosa* are largely aided by the ability of the bacteria to efficiently produce biofilms (9). These chronic biofilm-based infections are difficult, if not impossible, to eradicate due to physical, physiological, and genetic determinants that promote antibiotic tolerance, as well as mutational resistance caused by repeated antibiotic exposure. The emergence of multidrug-resistant (MDR) strains further enhances the threat level, with increased morbidity and mortality, as well as economic burdens, associated with these strains (10–13). The prevalence of MDR strains has increased over the past few decades, with certain geographic regions reporting 15% to 30% of *P. aeruginosa* strains as MDR (14–17). *P. aeruginosa* has been categorized as a critical clinical pathogen, an action stimulated by the clear unmet medical need for novel therapeutics that exhibit antipseudomonal potency toward biofilm-state and antibiotic-resistant strains (18).

S-type pyocins are chromosomally encoded proteinaceous bacteriocins produced by *P. aeruginosa* for intraspecies competition (19). These multimodular proteins contain at least two core structural components: an N-terminal domain that binds and initiates translocation across the outer membrane through a nutrient uptake receptor on the surface of *P. aeruginosa* and a C-terminal cytotoxic domain that exerts the lethal effect (e.g, endonuclease, lipid II degradation, or pore-forming activity). Pyocins are secreted from the producing cell as a binary protein complex consisting of the bacteriocin tightly bound to a smaller immunity protein that inhibits the lethal activity of the bacteriocin, thus preventing the producing strain from being harmed by its own pyocin prior to secretion (20). This complex dissociates once the N-terminal receptor-binding/translocation domain of the secreted bacteriocin binds to the target receptor expressed on the surface of a nearby *P. aeruginosa* cell.

Bacteriophage (phage) lysins are peptidoglycan hydrolases that are used to degrade the bacterial cell wall at the end of a phage infection cycle. This event promotes hypotonic lysis and consequently releases phage progeny from the phage-infected bacterium. Lysins derived from phage that infect Gram-positive bacteria are multimodular enzymes composed of an N-terminal catalytic domain linked to a C-terminal cell wall binding domain (CBD). The CBD binds with high affinity to a cell wall–associated carbohydrate to position the catalytic domain for peptidoglycan cleavage. Because Gram-negative bacteria have an outer membrane, lysins from a subset of phage infecting Gram-negative bacteria comprise a single catalytic domain equipped with a cationic segment that destabilizes the outer membrane structure, allowing for the effective release of phage progeny. However, when applied extrinsically as a recombinant protein, the ability of the cationic segment to disrupt the outer membrane of Gram-negative bacteria is inhibited in complex environments, such as serum. This prevents the catalytic domain from accessing its peptidoglycan substrate in the periplasm. Therefore, in their current form, the potential use of lysins as a human therapeutic for treating Gram-negative bacterial pathogens is restricted to superficial infections only (21, 22).

To overcome the inability of lysins to function in complex matrices against Gram-negative bacteria, we developed lysocins, which represent a new class of narrow-spectrum antimicrobials. These biologics use functional domains from S-type pyocins to deliver lysins across the outer membrane of *P. aeruginosa* in diverse environments. The lysin can then effectively cleave the peptidoglycan to cause rapid bacterial death (23). Translocation of lysocins across the outer membrane is mediated by TonB-dependent transporters, which are ubiquitously found in all Gram-negative bacteria (24). This process requires energy obtained from the proton motive force (PMF). The PMF is transferred to TonB-dependent transporters by the TonB-ExbB-ExbD motor complex in the inner membrane.

**Fig 1:**
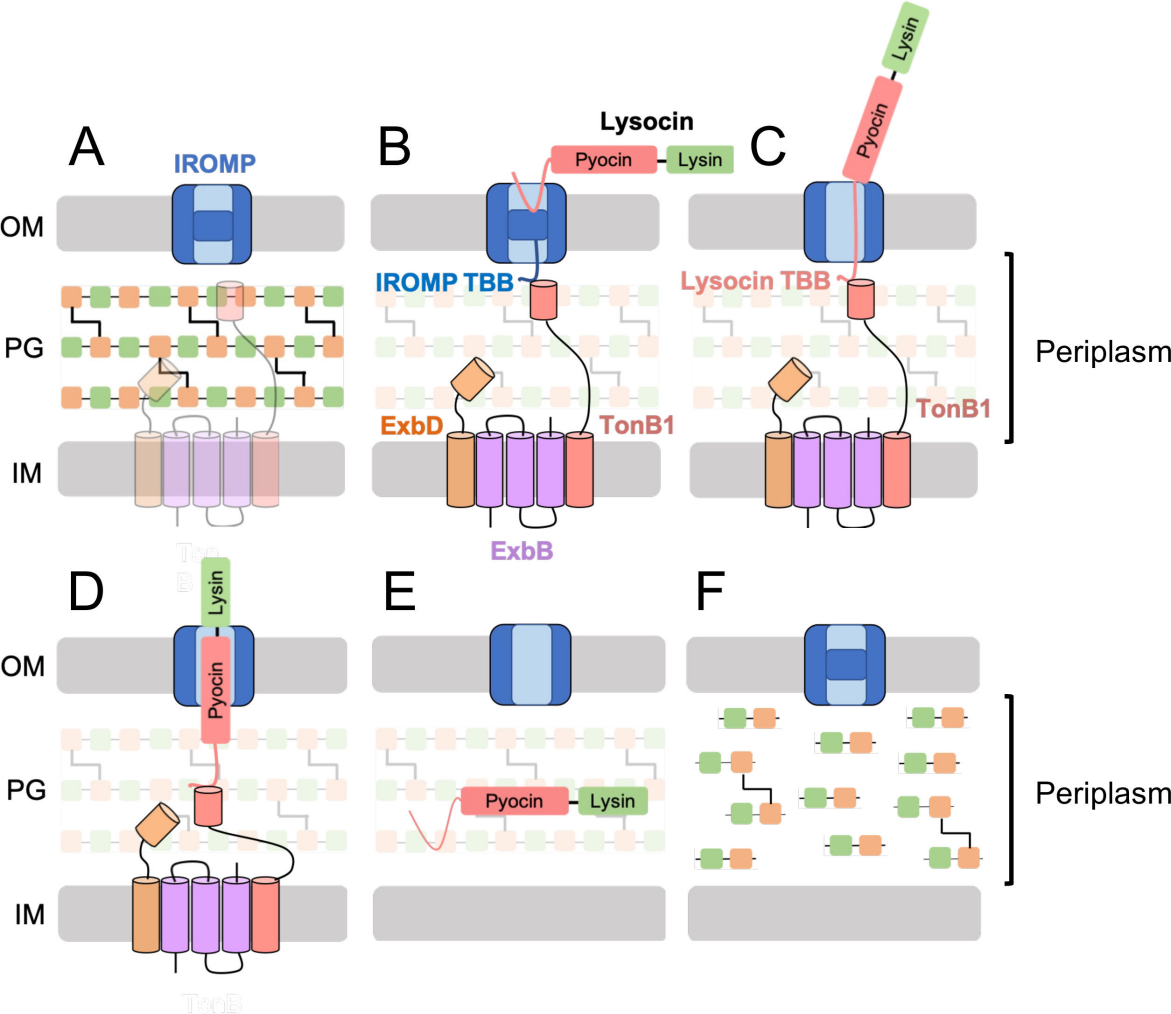
Proposed model of the antipseudomonal mechanism of action for lysocins. (**A**) In low-iron conditions, the gated, targeted iron-regulated outer membrane protein (IROMP) is expressed on the surface of *P. aeruginosa*. (**B**) When the lysocin is applied externally as a purified recombinant protein, the N-terminal unstructured region of the pyocin receptor-binding/translocation domain binds with high specificity to the IROMP. This interaction promotes an allosteric change within the receptor that exposes a periplasmic segment of the IROMP, specifically its TonB box (TBB) motif. The TBB binds the C-terminal domain of TonB1, which is a proton motive force (PMF)-linked transporter located in the inner membrane (IM). (**C**) Energy derived from the PMF transmitted from the TonB1-ExbB-ExbD complex in the IM results in the unfolding of the plug domain in the β barrel structure of the IROMP. The unstructured N-terminus of the lysocin can then diffuse through the resulting channel within the receptor, thereby allowing its own TBB to enter the periplasm and bind another nearby TonBI protein. (**D**) The formation of the lysocin-TonB1 complex provides the energy transduction from the PMF to facilitate the partial unfolding and translocation of the lysocin across the outer membrane (OM). (**E**) Upon successfully translocating to the periplasm, (**F**) the C-terminal lysin domain of the lysocin can cleave the peptidoglycan (PG) to promote membrane destabilization, cytoplasmic content leakage, and disruption of the PMF, consequently killing the bacterial cell.

In iron-limiting conditions, which occur in serum and other secretions, the N-terminal unstructured region constituted by the pyocin receptor-binding/translocation domain of the lysocin binds with high affinity to a gated iron-regulated outer membrane protein (IROMP), which functions as a TonB-dependent transporter on the surface of target *P. aeruginosa* (Fig. 1A and B). Receptor binding induces an allosteric change within the IROMP that exposes a short stretch of amino acids, known as the TonB box, in the periplasm. The TonB box is then bound by the C-terminal domain of the energy-transducing protein TonB1 in the inner membrane (Fig. 1B). Two additional inner membrane proteins, ExbB and ExbD, form a proton channel that provides the PMF to TonB1, consequently allowing for the TonB-dependent unfolding of the plug domain within the β barrel structure of the IROMP (Fig. 1C). Next, the N-terminal unstructured region of the lysocin can pass through the newly created channel within the IROMP, thereby allowing the lysocin to expose its own TonB box in the periplasm to another nearby TonB1 (Fig. 1C). Once the lysocin-TonB1 translocon is established, the second PMF-driven event occurs in which TonB1 provides the required energy needed to actively promote the partial unfolding and eventual import of the full-length lysocin across the outer membrane (Fig. 1D). Upon refolding after being transported to the periplasm (Fig. 1E), the C-terminal lysin domain of the lysocin weakens the cell wall by cleaving essential covalent bonds within the peptidoglycan structure (Fig. 1F). We hypothesized that this event promotes partial membrane destabilization and cytoplasmic leakage, which accordingly disrupts the PMF to kill the bacterial cell. In addition to outperforming standard-of-care antibiotics, other published lysocin hallmarks include bactericidal activity in human serum, potent antibiofilm properties, a narrow spectrum of antibacterial activity, and an absence of cytotoxicity toward human cells (23). Importantly, we have demonstrated the ability of parenteral dosing with lysocins to eliminate clinically relevant *P. aeruginosa* in a lethal model of murine bacteremia.

Despite this success, the primary design challenge in lysocin development is overcoming the strain specificity conferred by the receptor-binding/translocation domain. To date, the proof-of-concept lysocin, PyS2-GN4, only targets *P. aeruginosa* strains that express the ferripyoverdine A type I (FpvAI) receptor due to the specificity of the pyocin S2 (PyS2) receptor-binding/translocation domain segment of the lysocin (23). In general, the species of *P. aeruginosa* can be collectively divided into three distinct siderovars based on the FpvA receptor type expressed: specifically, those expressing either type I (FpvAI), type II (FpvAII), or type III (FpvAIII) receptors (25). A genotypic analysis of *P. aeruginosa* revealed that the distribution frequency of all three FpvA receptor types is similar among certain populations of *P. aeruginosa* clinical isolates (26). For this reason, the specificity of lysocins needs to be broadened to cover most, if not all, clinical isolates of *P. aeruginosa*.

In this work, two second-generation lysocin candidates were designed with the goal of expanding *P. aeruginosa* strain specificity. Unlike the PyS2-GN4 lysocin, which comprises three pyocin domains, these new lysocins consisted of a *Pseudomonas* lysin fused to a single pyocin domain that dually functions to bind and promote translocation through highly conserved IROMPs of *P. aeruginosa*. The receptor-binding/translocation domain candidates we selected originate from either pyocin S5 (PyS5), which targets the conserved ferric pyochelin transporter (FptA), or pyocin G (PyG), which targets the conserved hemin uptake receptor (Hur) (27, 28). We found that the lead lysocin candidate that exhibited the most potent antipseudomonal activity was PyS5-I-GN4. This lysocin was further characterized *in vitro* and then ultimately *in vivo* using an acute neutropenic murine lung infection model of disease and was found to be most effective in controlling nearly all of the MDR clinical isolates of *P. aeruginosa* tested.

## RESULTS

### Antipseudomonal effect of removing dispensable functional domains from lysocin PyS2-GN4

**Fig 2:**
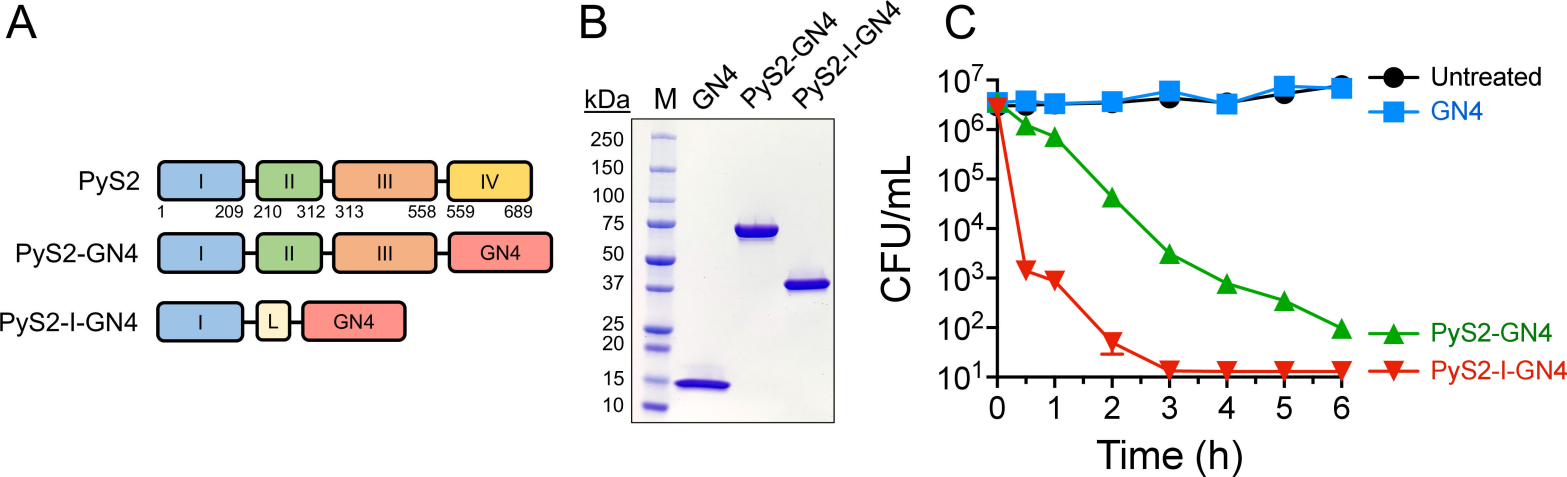
Effect of domain composition on the antipseudomonal properties of PyS2-derived lysocins. (**A**) For the proof-of-concept lysocin PyS2-GN4, domains I, II, and III of PyS2 were fused to the GN4 lysin. Domain dispensability was evaluated by removing domains II and III from the lysocin. The resulting truncated PyS2-I-GN4 lysocin comprised domain I fused to the GN4 lysin using a GSx3 linker (annotated as L). (**B**) The purity of the GN4 lysin (16 kDa), including the PyS2-GN4 (76 kDa) and PyS2-I-GN4 (40 kDa) lysocins, was assessed using sodium dodecyl sulfate polyacrylamide gel electrophoresis. (**C**) A 6-hour time-kill assay was used to measure the killing kinetics of PyS2-GN4 and PyS2-I-GN4 at 0.5 µM against *P. aeruginosa* strain 453 in iron-depleted growth medium at 37°C, with bacterial viability assessed at various time points. Untreated and GN4-treated bacteria served as controls. The CFU/mL concentration of viable bacteria was quantitated following serial dilution and plating on CAA agar. The limit of detection was 10 CFU/ml. Error bars correspond to the ± standard error of the mean of triplicate biological replicates.

PyS2 consists of four functional domains. Domain I (amino acids [aa] 1–209) is a receptor-binding/translocation domain; domain II (aa 210–312) is an α-helical domain that binds to the common polysaccharide antigen of lipopolysaccharide; domain III (aa 313–558) is homologous to colicins of *Escherichia coli*; and domain IV (aa 559–689) functions as an endonuclease (29–31). Our proof-of-concept lysocin PyS2-GN4 was engineered by fusing domains I, II, and III of PyS2 to the GN4 lysin, which is a 144 aa muramidase from phage PAJU2 of *P. aeruginosa* (Fig. 2A) (23). To evaluate the dispensability of domains II and III of PyS2, a truncated lysocin construct was designed, termed PyS2-I-GN4, consisting of only domain I fused via a GSx3 linker to the GN4 lysin (Fig. 2A).

The antipseudomonal killing kinetics of purified PyS2-I-GN4 (Fig. 2B) were compared to the full-length (three pyocin domains) PyS2-GN4 lysocin at equal molar concentrations (0.5 µM) using a 6-hour time-kill assay. For these experiments, untreated and GN4-treated *P. aeruginosa* were used as negative controls for antipseudomonal activity. At 30 min, PyS2-I-GN4 was bactericidal (3.3-log reduction), which is defined as ≥3-log reduction with respect to the untreated control (Fig. 2C). By 3 h, PyS2-I-GN4 decreased bacterial counts by >5 logs to below the limit of detection (LOD), which was 10 colony-forming units per milliliter (CFU/mL). Conversely, the full-length PyS2-GN4 lysocin required 3 h to achieve bactericidal activity (≥3-log reduction) and was incapable of reducing pseudomonal viability to the LOD over the 6-hour experiment. These results clearly indicate that removing dispensable domains II and III increased killing efficiency.

### Expanding *P. aeruginosa* strain coverage by engineering second-generation lysocins

As mentioned, domain I of PyS2 binds and initiates translocation through FpvAI, which is expressed on the surface of only one-third of all *P. aeruginosa* clinical isolates. Accordingly, the specificity of the PyS2-derived lysocins for FpvAI-typed *P. aeruginosa* strains was confirmed using a modified broth microdilution assay. For these experiments, the minimum inhibitory concentration (MIC) of PyS2-I-GN4 was measured against 13 FpvA-typed clinical isolates of *P. aeruginosa*. As expected, this lysocin exhibited antipseudomonal activity against all FpvAI-typed strains, with MIC values of ≤8 µg/mL (Table 1). However, PyS2-I-GN4 was ineffective toward *P. aeruginosa* strains that express FpvAII or FpvAIII.

**TABLE 1:** MIC of the PyS2-I-GN4 lysocin against FpvA-typed *P. aeruginosa* clinical isolates** **

*P. aeruginosa* strain	FpvA type	MIC (μg/mL)
PAO1	I	4
453	I	0.25
AR465	I	4
AR469	I	8
AR470	I	8
AR474	I	4
445	II	>256
448	II	>256
451	II	>256
AR468	II	>256
443	III	>256
450	III	>256
471	III	>256

**Fig 3:**
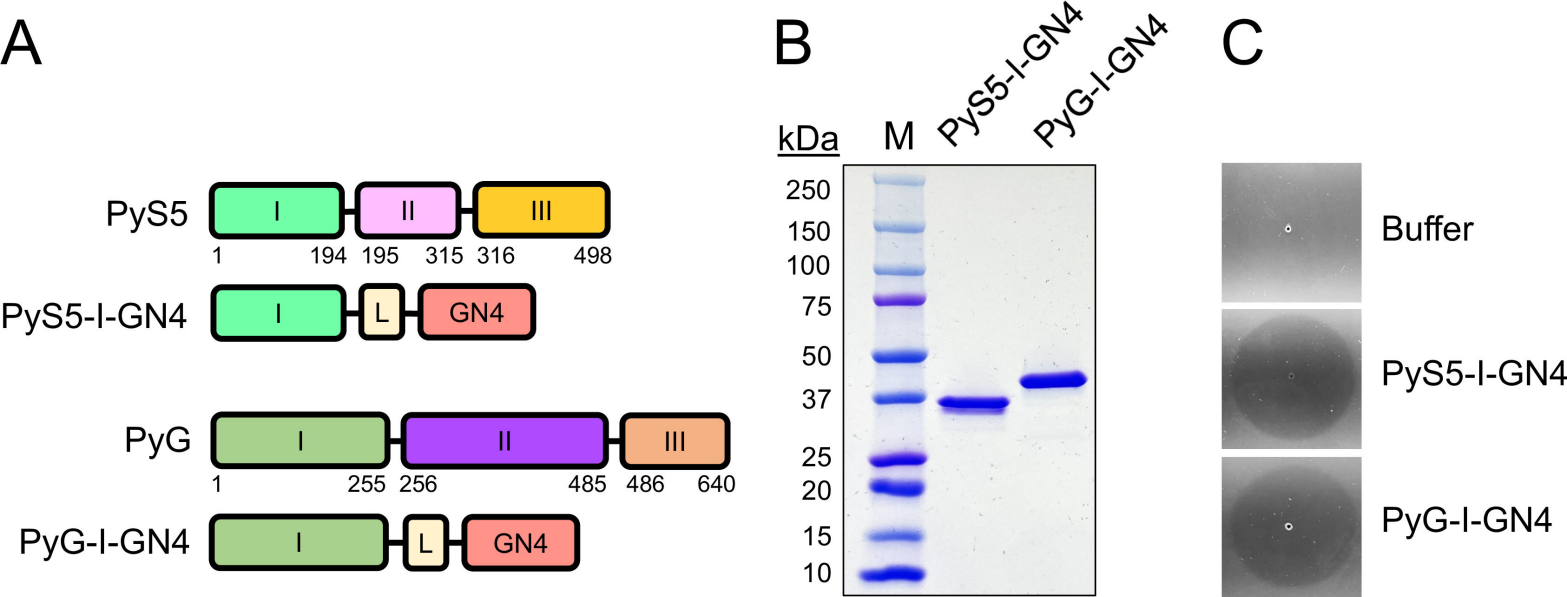
Construct design, purification, and muralytic activity of second-generation lysocins. (**A**) PyS5 and PyG bind and translocate through the highly conserved *P. aeruginosa* receptors FptA and Hur, respectively. Lysocins with broadened antipseudomonal activity were designed by fusing domain I of either PyS5 or PyG, which serves as the receptor-binding/translocation domain, to the GN4 lysin using a GSx3 linker (annotated as L). (**B**) The purity of the PyS5-I-GN4 (39 kDa) and PyG-I-GN4 lysocins (44 kDa) was determined using sodium dodecyl sulfate polyacrylamide gel electrophoresis. (**C**) The muralytic activity of PyS5-I-GN4 and PyG-I-GN4 was assayed by spotting 25 pmol of each purified protein on autoclaved *P. aeruginosa*. Buffer was spotted as a negative control for muralytic activity. Clearing zones observed after an overnight incubation at 37°C correspond to peptidoglycan cleavage.

Strain coverage could presumably be expanded by designing lysocins with pyocin components that bind to and translocate through more conserved IROMPs. To test this hypothesis, second-generation lysocins were designed with a pyocin receptor-binding/translocation domain originating from either PyS5 or PyG. PyS5 consists of 498 aa organized into three domains: (i) domain I (aa 1–194) binds and initiates translocation through FptA; (ii) domain II (aa 195–315) binds to the common polysaccharide antigen to promote accumulation of the pyocin on the bacterial surface; and (iii) domain III (aa 316–498) damages the bacterial membrane by means of pore formation (32) (Fig. 3A). PyG consists of 640 aa organized into three domains: (i) domain I (aa 1–255) binds and initiates translocation through Hur; (ii) domain II (aa 256–485) is the inner membrane translocation domain; and (iii) domain III (aa 486–640) functions as an endonuclease (27, 33).

Our preliminary studies revealed that omitting dispensable pyocin domains beyond domain I increased the antipseudomonal potency of the PyS2-derived lysocins (Fig. 2C). Thus, second-generation lysocins were designed consisting of domain I from either PyS5 or PyG fused via a GSx3 linker to the GN4 lysin (Fig. 3A). The resulting purified PyS5-I-GN4 and PyG-I-GN4 lysocins (Fig. 3B) each displayed muralytic activity when spotted at equal molar concentrations on autoclaved *Pseudomonas* (Fig. 3C), confirming that the GN4 lysin retains enzymatic activity when fused to domain I of either pyocin.

*Pseudomonas* strain specificity was evaluated for PyS2-I-GN4 (control), PyS5-I-GN4, and PyG-I-GN4 by determining the MIC of each lysocin using a collection of 25 MDR respiratory clinical isolates, including five CF isolates, and 15 MDR blood and wound clinical isolates of *P. aeruginosa*. The *P. aeruginosa* reference strain PAO1 was also included in the collection of blood and wound isolates. For the respiratory isolates, PyS2-I-GN4, PyS5-I-GN4, and PyG-I-GN4 were effective against 44% (11/25 strains), 92% (23/25 strains), and 72% (18/25 strains) of the strains, respectively (Table 2). For the blood and wound isolates, 38% (6/16 strains), 100% (16/16 strains), and 38% (6/16 strains) of the strains were sensitive to PyS2-I-GN4, PyS5-I-GN4, and PyG-I-GN4, respectively (Table 3). Combining the results from blood, wound, and respiratory strains, only 41% strain coverage was observed for PyS2-I-GN4, while 95% and 59% coverage was observed for PyS5-I-GN4 and PyG-I-GN4 lysocins, respectively.

**TABLE 2:** MIC of PyS2-I-GN4 and second-generation lysocins toward MDR respiratory clinical isolates of *P. aeruginosa*

	MIC (μg/mL)		
*P. aeruginosa* Strain	PyS2-I-GN4	PyS5-I-GN4	PyG-I-GN4
NR-51515	>256	4	8
NR-51519	4	4	4
NR-51524	>256	8	8
NR-51528	>256	8	8
NR-51537	4	4	4
NR-51540	>256	8	8
NR-51570	>256	8	>256
NR-51571	4	8	8
NR-51573	8	8	>256
NR-51577	4	4	>256
NR-51580	>256	4	8
NR-51584	>256	8	8
NR-51587	>256	4	4
NR-51589	>256	16	8
NR-51594	8	>256	>256
NR-51597	4	16	16
NR-51598	>256	8	>256
NR-51603	>256	>256	>256
NR-51604	>256	8	>256
NR-51614	4	4	8
RH8	>256	4	8
RH13	64	8	4
RH14	>256	4	8
RH22	2	4	4
RH24	2	2	4

**TABLE 3:** MIC of PyS2-I-GN4 and second-generation lysocins toward *P. aeruginosa* strain PAO1 and MDR blood and wound clinical isolates

	MIC (μg/mL)		
*P. aeruginosa* Strain	PyS2-I-GN4	PyS5-I-GN4	PyG-I-GN4
PAO1	4	8	8
NR-51516	>256	4	8
NR-51517	>256	8	8
NR-51525	8	8	>256
NR-51530	>256	32	>256
NR-51536	>256	8	>256
NR-51546	>256	16	>256
NR-51549	16	16	>256
NR-51556	4	8	>256
NR-51557	4	8	8
NR-51565	>256	8	>256
NR-51568	32	8	4
NR-51569	>256	8	8
NR-51574	>256	8	>256
NR-51579	>256	16	>256
NR-51593	>256	8	>256

### Antipseudomonal potency of second-generation lysocins

**Fig 4:**
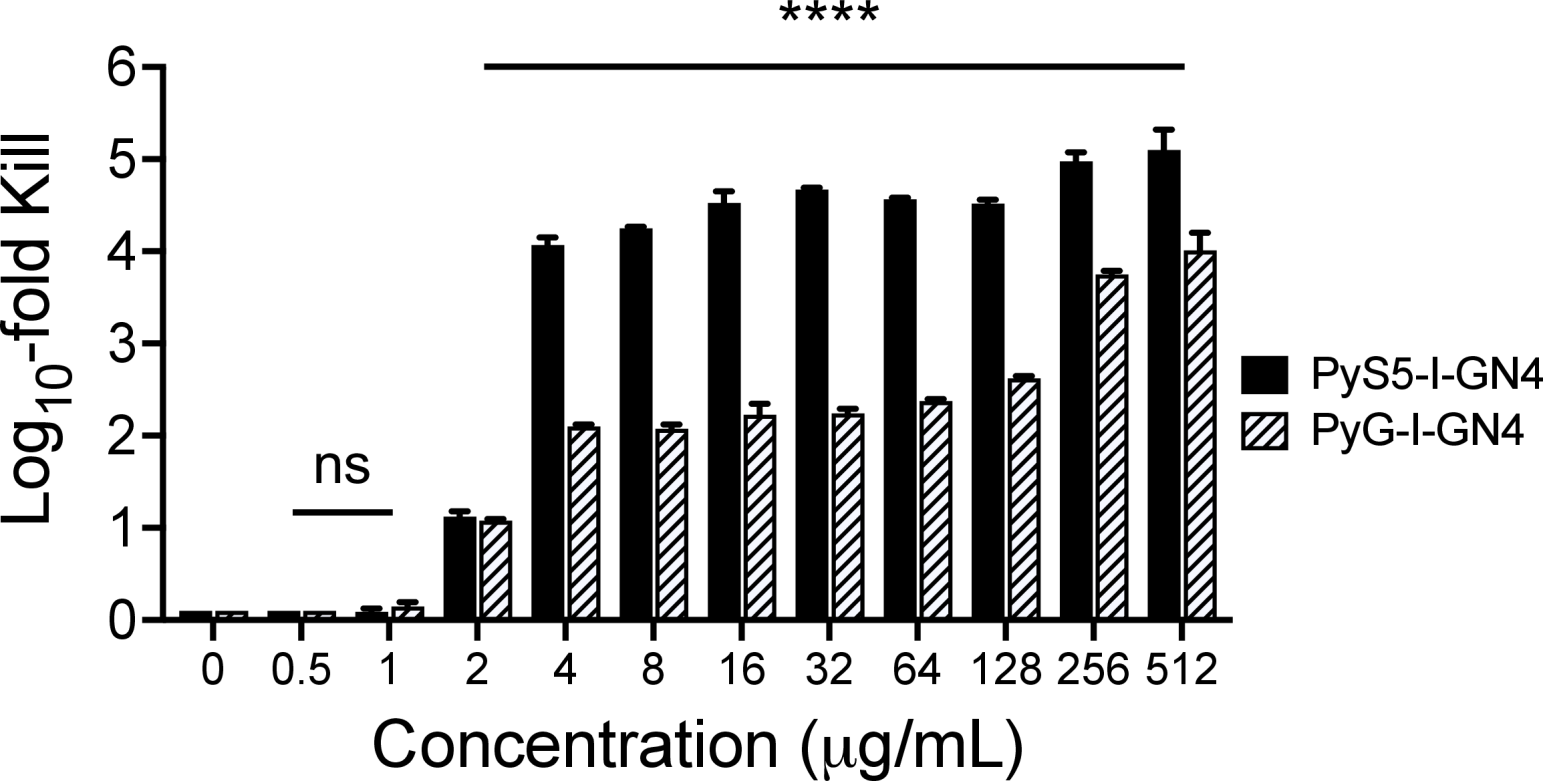
Antipseudomonal potency of PyS5-I-GN4 and PyG-I-GN4. Dose-response killing assays were used to compare the antipseudomonal potency of PyS5-I-GN4 and PyG-I-GN4. *P. aeruginosa* strain PAO1 was incubated with each lysocin at final concentrations ranging from 0.5 to 512 µg/mL (~10 nM to 13 µM) for 4 h at 37°C in iron-depleted growth medium. Bacteria absent lysocin served as negative controls for antipseudomonal activity. Bacterial viability was enumerated by serial dilution and plating on CAA agar. Data is depicted as the log_10_-fold reduction in CFU/mL with respect to the untreated control. The limit of detection was 10 CFU/mL. Error bars correspond to the ± standard error of the mean of triplicate biological replicates. Significance levels were determined using an unpaired Student *t*-test. ns, not significant, ****P < 0.0001.

The antipseudomonal killing efficiency of PyS5-I-GN4 and PyG-I-GN4 was measured using a 4-hour dose-response killing assay. For these experiments, the *P. aeruginosa* strain PAO1 was treated with each lysocin at concentrations ranging from 0.5 to 512 µg/mL (~10 nM to 13 µM) in iron-depleted growth medium. A log_10_-fold reduction in bacterial viability by PyS5-I-GN4 was observed at concentrations as low as 2 µg/mL (51 nM) (Fig. 4). The lysocin reduced bactrial counts by at least 4 logs at ≥4 µg/mL (103 nM). Although PyG-I-GN4 was also capable of log_10_-fold reductions in bacteria at concentrations as low as 2 µg/mL (45 nM), it consistently showed less than 1–3-log reduction when compared to PyS5-I-GN4 at equal concentrations. Compared to PyG-I-GN4, the superior antipseudomonal potency (Fig. 4) and expanded strain coverage (Tables 2 and 3) displayed by PyS5-I-GN4 resulted in this particular lysocin being the focus of subsequent experiments.

### Bacterial species targeted by PyS5-I-GN4

Considering that PyS5-I-GN4 requires the FptA receptor for outer membrane translocation, it was anticipated that the lysocin would have a very narrow spectrum and exhibit antibacterial activity exclusively toward *P. aeruginosa*, since this particular bacterial species uniquely expresses FptA. To confirm this, the MIC of PyS5-I-GN4 was tested against a collection of Gram-positive and Gram-negative bacterial pathogens. Compared to the *P. aeruginosa* control (MIC 4 µg/mL), PyS5-I-GN4 displayed no antibacterial activity against the 12 different bacterial species tested, including five different *Pseudomonas* species (Table S1). Thus, as expected, the antibacterial effects of PyS5-I-GN4 appear to be specific to *P. aeruginosa* only.

### Targeted drug delivery of additional lysins to *P. aeruginosa* using domain I of PyS5

**Fig 5:**
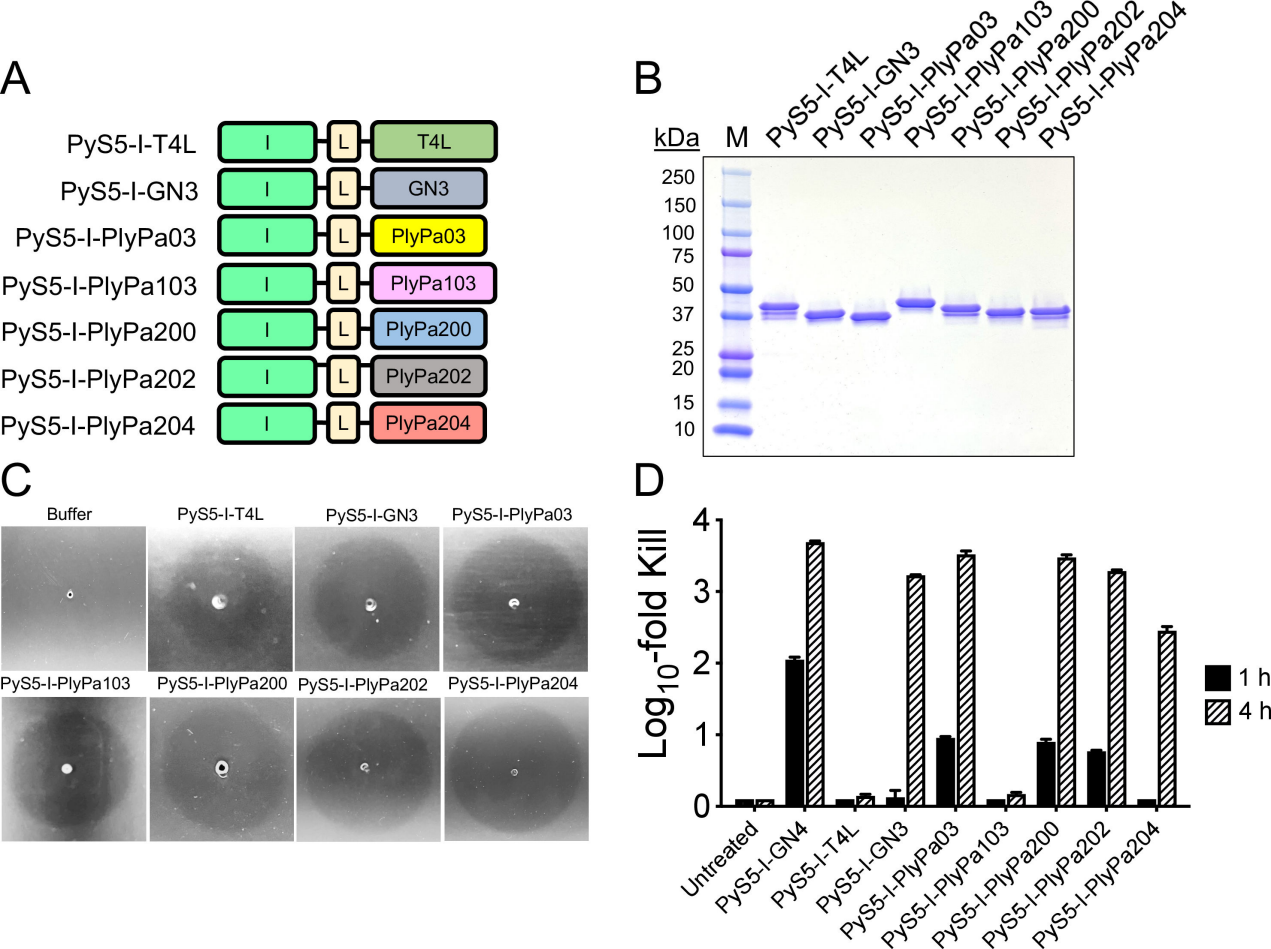
Using domain I of PyS5 to deliver additional lysins across the outer membrane of *P. aeruginosa*. (**A**) A collection of PyS5-derived lysocins was designed that comprised domain I of PyS5 fused using a GSx3 linker (annotated as L) to the lysin candidates T4L, GN3, PlyPa03, PlyPa103, PlyPa200, PlyPa202, and PlyPa204. (**B**) The purity of the PyS5-I-T4L (42 kDa), PyS5-I-GN3 (39 kDa), PyS5-I-PlyPa03 (39 kDa), PyS5-I-PlyPa103 (42 kDa), PyS5-I-PlyPa200 (39 kDa), PyS5-I-PlyPa202 (39 kDa), and PyS5-I-PlyPa204 lysocins (39 kDa) was visualized by sodium dodecyl sulfate polyacrylamide gel electrophoresis. (**C**) The muralytic activity of each lysocin was qualitatively assessed by spotting 25 pmol of each purified protein on autoclaved *P. aeruginosa*. Buffer was spotted as a negative control for muralytic activity. Clearing zones observed after an overnight incubation at 37°C are indicative of peptidoglycan cleavage. (**D**) A 4-hour time-kill assay was utilized to measure the killing efficiency of each lysocin at 0.5 µM against *P. aeruginosa* strain PAO1 in iron-depleted growth medium at 37°C. Bacterial viability was assessed at 1 and 4 h by serial dilution and plating on CAA agar. *Pseudomonas* treated with PyS5-I-GN4 were used as a positive control for antipseudomonal activity, whereas bacteria without lysocin treatment represented negative controls. Data are shown as the log_10_-fold reduction in CFU/mL relative to untreated control. The limit of detection was 10 CFU/mL. Error bars correspond to the ± standard error of the mean of triplicate biological replicates.

Additional lysocin constructs were designed to evaluate the broad applicability of domain I of PyS5 in delivering other lysins across the outer membrane of *P. aeruginosa*. Lysin candidates tested included the *E. coli* T4 lysozyme (T4L) and the *Pseudomonas* lysins GN3, PlyPa03, PlyPa103, PlyPa200, PlyPa202, and PlyPa204. Each of these lysin candidates comprises a single catalytic domain that is predicted to function as a muramidase. Results from a sequence alignment with the GN4 lysin revealed that the T4L, GN3, PlyPa03, PlyPa103, PlyPa200, PlyPa202, and PlyPa204 lysins share 20%, 74%, 94%, 8%, 76%, 76%, and 74% sequence identity, respectively. Domain I of PyS5 was fused to each of the aforementioned lysins using a GSx3 linker (Fig. 5A).

Spotting the purified lysocins (Fig. 5B) on autoclaved *P. aeruginosa* produced observable clearing zones, indicating that each lysin retains muralytic activity when fused to domain I of PyS5 (Fig. 5C). Next, the antipseudomonal potency of each lysocin at 0.5 µM (~20 µg/mL) was measured against *P. aeruginosa* strain PAO1 using a 4-hour time-kill assay. PyS5-I-GN4 and untreated bacteria were used as respective positive and negative controls. At 1 h, the PyS5-I-GN4 control decreased bacterial viability by 2 logs, while PyS5-I-PlyPa03, PyS5-I-PlyPa200, and PyS5-I-PlyPa202 exhibited about a 1-log reduction (Fig. 5D). However, similar to the PyS5-I-GN4 control, bactericidal activity (≥3 log reduction) was observed at 4 h for PyS5-I-GN3, PyS5-I-PlyPa03, PyS5-I-PlyPa200, and PyS5-I-PlyPa202. PyS5-I-PlyPa204 reduced bacterial counts by 2.5 logs at 4 h. Minimal antipseudomonal activity was detected for PyS5-I-T4L and PyS5-I-PlyPa103. These findings suggest that domain I of PyS5 can be broadly utilized to deliver other, but not all, lysins across the outer membrane of *P. aeruginosa*, while retaining antipseudomonal potency comparable to that of PyS5-I-GN4.

### *In vitro* antipseudomonal properties of PyS5-I-GN4 in complex matrices

**Fig 6:**
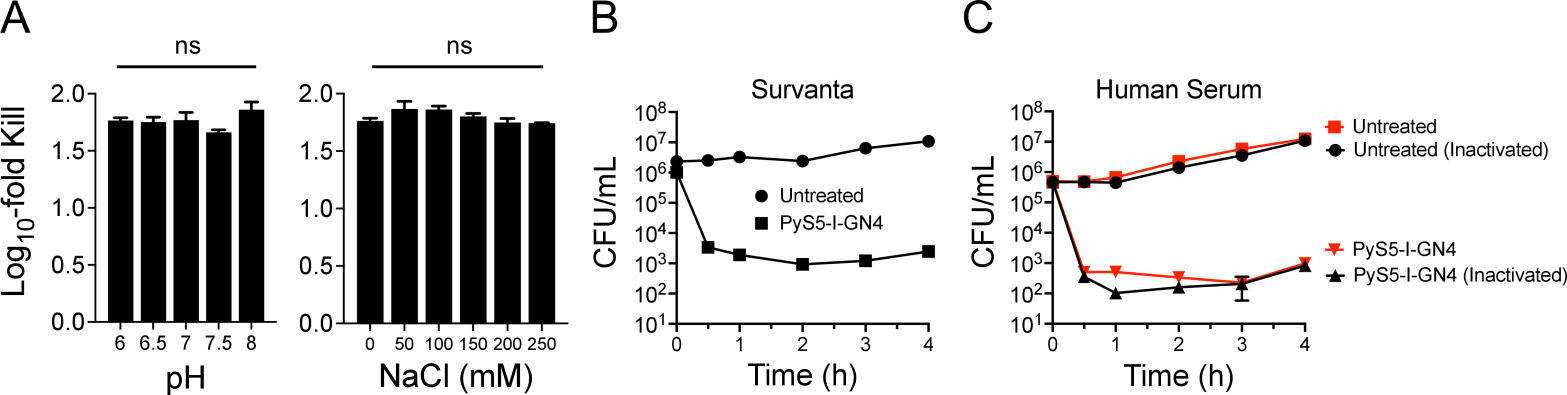
Biochemical characteristics and *in vitro* bactericidal activity of PyS5-I-GN4 in lung surfactant and serum. (**A**) The effect of pH (left) and salt (right) on the antipseudomonal activity of PyS5-I-GN4 was evaluated by treating *P. aeruginosa* strain PAO1 with lysocin at 0.5 µM in either 50 mM sodium phosphate (pH 6.0 to 8.0) or 20 mM sodium phosphate (pH 7.3) supplemented with 0 to 250 mM NaCl, respectively, for 4 h at 37°C. An untreated control was included for each pH and salt concentration assayed. Bacterial viability was assessed following serial dilution and plating on CAA agar. Data is depicted as the log_10_-fold reduction in CFU/mL when compared to the untreated control. *P*-values were calculated using a one-way ANOVA Brown-Forsythe test. ns, not significant. A 4-hour time-kill assay was utilized to elucidate the antipseudomonal killing kinetics of PyS5-I-GN4 at 0.5 µM in either (**B**) 50% (vol/vol) lung surfactant (Survanta) or (**C**) human serum (red) and heat-inactivated human serum (black). Bacterial viability at 37°C was quantitated at various time points by serial dilution and plating on CAA agar. Bacteria in the absence of lysocin were used as untreated controls. The limit of detection was 10 CFU/mL. Error bars correspond to the ± standard error of the mean of triplicate biological replicates.

*P. aeruginosa* has consistently been a leading cause of nosocomial infections, especially respiratory and bloodstream infections. Among Gram-negative bacteria, *P. aeruginosa* is the predominant cause of nosocomial pneumonia and the third most common cause of bloodstream infections (3, 34). Accordingly, the antipseudomonal activity of PyS5-I-GN4 at 0.5 µM was assayed at physiological pH and sodium chloride concentrations observed in the airway surface liquid of the lungs (~pH 7.3, 120 mM NaCl) and in serum (pH 7.4, 140 mM NaCl) (35, 36). Findings from a 4-hour ing assay revealed that lysocin activity was unaffected when treating *P. aeruginosa* in sodium phosphate buffer at pH values of 6.0 to 8.0 (Fig. 6A, left) and at salt concentrations ranging from 0 to 250 mM NaCl (Fig. 6A, right).

To expand on these findings, the antipseudomonal killing kinetics of PyS5-I-GN4 were subsequently measured in the presence of either lung surfactant or human serum using a 4-hour time-kill analysis. PyS5-I-GN4 rapidly killed *Pseudomonas* in both beractant (Survanta) (Fig. 6B) and human serum (Fig. 6C). The lysocin at 0.5 µM was bactericidal (3.2-log reduction) within 1 h in beractant (Fig. 6B), and was also bactericidal within 30 min in human serum (3.0 log reduction) (Fig. 6C, red), compared to the untreated controls. Heat-inactivated human serum was also tested in order to elucidate the effect, if any, that complement and other heat-labile serum proteins have on lysocin activity. PyS5-I-GN4 retained high antipseudomonal potency in inactivated serum, with bactericidal activity observed within 30 min (3.1-log reduction) (Fig. 6C, black). Like PyS2-derived lysocins (23), PyS5-derived lysocins were bactericidal toward *P. aeruginosa* in complex matrices.

### Antibiofilm properties of PyS5-I-GN4

**Fig 7:**
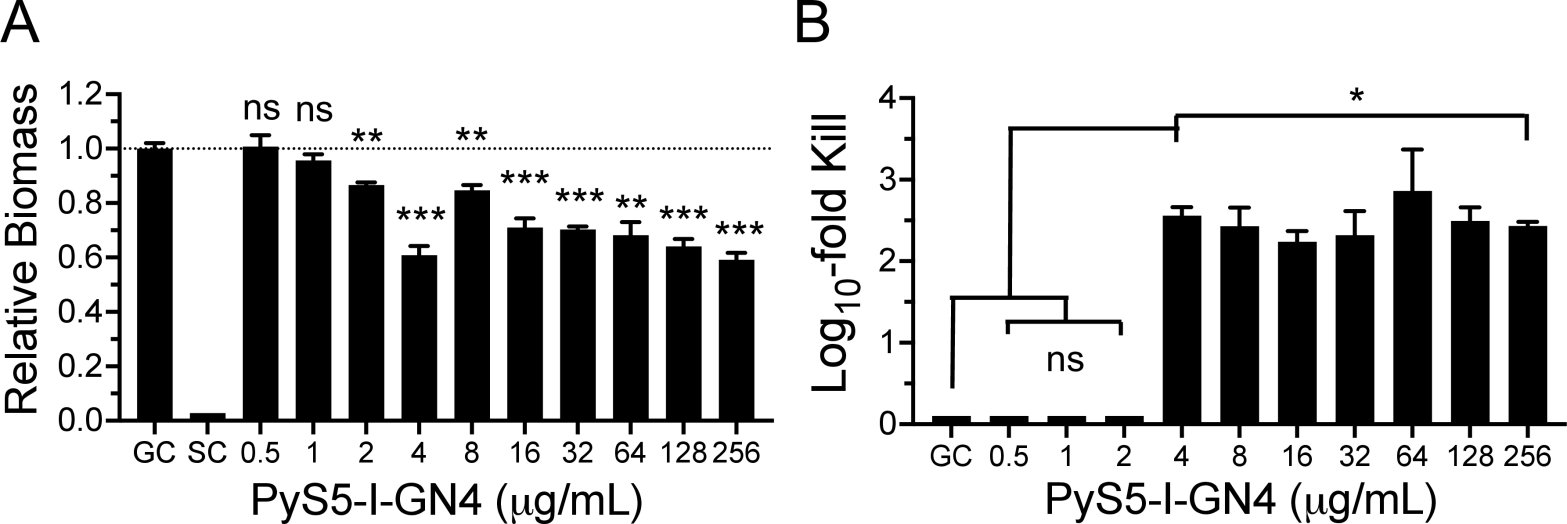
*In vitro* antibiofilm activity of PyS5-I-GN4. *P. aeruginosa* strain PAO1 biofilms were grown on plastic pegs of an MBEC Assay Biofilm Inoculator in tryptic soy broth with 0.2% glucose for 24 h at 37°C with aeration. The mature biofilms were washed with buffer and treated statically overnight with PyS5-I-GN4 at 0.5 to 256 µg/mL (13 nM to 7 µM) in iron-depleted CAA medium with 0.2% glucose. Growth controls (GC), which consisted of biofilms grown in the absence of lysocin, and sterility controls (SC), which constituted growth medium only, were included. (**A**) For quantitating biofilm biomass, each biofilm was initially stained with 0.05% (wt/vol) crystal violet. Following a wash step, residual crystal violet was solubilized with 33% (vol/vol) acetic acid. Each sample was then transferred to a 96-well microtiter plate and subsequently assayed at an OD_600nm_ using a microplate reader. Data was normalized to the biofilm biomass of the GC. (**B**) For measuring bacterial viability, biofilms from an independent MBEC plate were homogenized in 20 mM sodium phosphate, pH 7.0, using a water bath sonicator. The contents of each sample were serially diluted and plated on CAA agar. Data is shown as the log_10_-fold reduction in CFU/mL with respect to the GC. The limit of detection was 10 CFU/mL. Error bars correspond to the ± standard error of the mean of triplicate biological replicates. *P*-values were calculated using an unpaired Student *t*-test. ns, not significant, *P < 0.05, **P < 0.01, ***P < 0.001.

The ability of PyS5-I-GN4 to eradicate biofilm structures and kill bacteria within these microenvironments was measured *in vitro*. Using an MBEC Assay Biofilm Inoculator, 24-hour mature *P. aeruginosa* biofilms were treated overnight with varying concentrations of lysocin. Biofilm biomass and bacterial viability were then measured. Compared to the growth control, PyS5-I-GN4 at ≥4 µg/mL (103 nM) reduced biofilm biomass between 30% and 40% (Fig. 7A) and decreased bacterial viability between 2 and 3 logs (Fig. 7B). These results highlight the ability of PyS5-I-GN4 to both disrupt biofilms and kill bacteria constituted within these microenvironments.

### Lysocin synergy with standard-of-care antibiotics

Despite its antipseudomonal potency when used alone, PyS5-I-GN4 could be most effective as a human therapeutic when used in combination with standard-of-care antibiotics. To this end, a checkerboard analysis was used to evaluate synergy between PyS5-I-GN4 and several clinically relevant antipseudomonal antibiotics. Antibiotics tested include colistin sulfate (lipopeptide), cefepime (cephalosporin), levofloxacin (fluoroquinolone), meropenem (carbapenem), piperacillin/tazobactam (penicillin), and tobramycin (aminoglycoside). These antibiotics collectively represent most of the major antipseudomonal antibiotic classes. The Fractional Inhibitory Concentration Index (FICI) of each lysocin and antibiotic combination was quantitated to determine if the potency of the two drugs is synergistic (≤0.5), additive (>0.5 and ≤1.0), indifferent (>1.0 and ≤4), or antagonistic (>4). PyS5-I-GN4 combined with colistin yielded an FICI value of 1, indicating an additive effect, while using the lysocin in combination with the remaining antibiotics resulted in an indifferent effect (FICI values > 1 and ≤4).

### Cytotoxicity toward eukaryotic cells and endotoxin release

**Fig 8:**
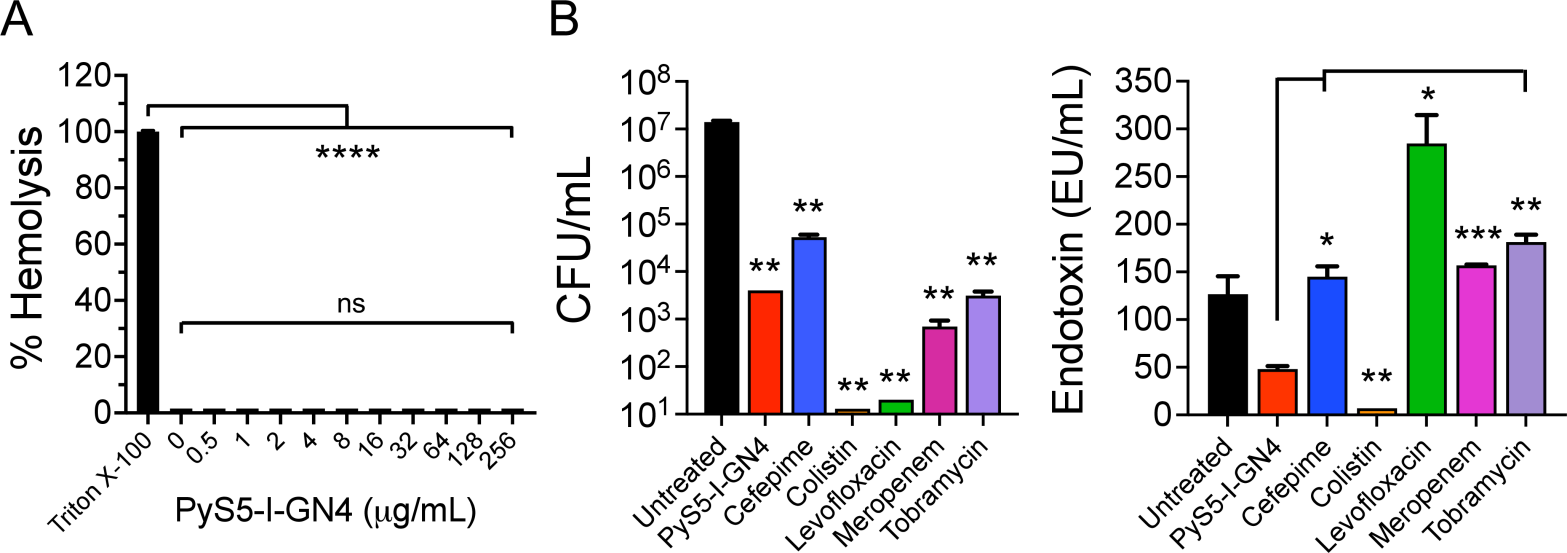
PyS5-I-GN4 cytotoxicity toward human red blood cells and endotoxin release. (**A**) The hemolytic properties of PyS5-I-GN4 were assayed by incubating human red blood cells obtained from healthy adult donors with lysocin concentrations ranging from 0.5 to 256 µg/mL (13 nM to 7 µM) in PBS, pH 7.4, for 4 h at 37°C with 5% CO_2_. After removing intact red blood cells, the relative concentration of hemoglobin released in the sample supernatant was quantitated by measuring the OD_405nm_ using a microplate reader. Blood cells incubated with either 0.1% Triton X-100 or buffer only represented positive and negative controls for hemolysis, respectively. Error bars correspond to the ± standard error of the mean of triplicate biological replicates. *P*-values were calculated using a one-way ANOVA Brown-Forsythe test. ns, not significant, ****P < 0.0001. (**B**) (Left) The bactericidal activity of PyS5-I-GN4 and standard-of-care antibiotics used clinically for treating pseudomonal lung infections in cystic fibrosis patients was quantitated toward *P. aeruginosa*. For this killing assay, *P. aeruginosa* strain PAO1 was treated with 5× MIC concentrations of either PyS5-I-GN4, cefepime, colistin, levofloxacin, meropenem, or tobramycin for 4 h at 37°C in iron-depleted growth medium. Bacterial viability was measured by serial dilution and plating on CAA agar. Untreated bacteria represented negative controls for bactericidal activity. The limit of detection was 10 CFU/mL. (Right) In parallel, endotoxin levels in the filtered supernatants from each sample used in the aforementioned killing assay were quantitated using a ToxinSensor Chromogenic LAL Endotoxin Assay Kit. The data is presented as endotoxin units (EU) per mL (EU/mL). Error bars correspond to the ± standard error of the mean of duplicate biological replicates. *P*-values were calculated using an unpaired Student *t*-test. ns, not significant, *P < 0.05, **P < 0.01, ***P < 0.001.

PyS5-I-GN4 is not membrane-acting and targets an outer membrane receptor (FptA) and substrate (peptidoglycan) only found in prokaryotes. Therefore, we anticipated that the lysocin would lack cytotoxicity toward eukaryotic cells. To confirm this, PyS5-I-GN4 cytotoxicity was measured toward human red blood cells. The cells were incubated for 4 h at 37°C with PyS5-I-GN4 at 0.5 to 256 µg/mL (~13 nM to 7 µM). Blood cells incubated with Triton X-100 or phosphate buffered saline (PBS) served as positive and negative control for hemolysis, respectively. Following treatment, intact blood cells were subsequently removed by low-speed centrifugation, and the relative amount of hemoglobin released was spectrophotometrically assayed. Similar to the PBS-treated negative control group, data obtained from these experiments showed that the lysocin was not cytotoxic toward human red blood cells at all concentrations tested (Fig. 8A).

*P. aeruginosa* endotoxin may contribute to the pathogenesis of CF by promoting hypersecretion of mucus and IL-8 production in tracheal epithelial cells (37–39). Due to the potential adverse effects of liberated endotoxin on patient morbidity, the amount of endotoxin released by *P. aeruginosa* treated with PyS5-I-GN4 was measured and compared to bacteria treated with antipseudomonal antibiotics. For these studies, *P. aeruginosa* strain PAO1 was treated with 5× MIC concentrations of either PyS5-I-GN4 (MIC 4 µg/mL), cefepime (MIC 2 µg/mL), colistin (MIC 2 µg/mL), levofloxacin (MIC 1 µg/mL), meropenem (MIC 1 µg/mL), or tobramycin (MIC 0.25 µg/mL). After 4 h at 37°C, both bacterial viability and endotoxin release were quantitated. As expected, each antimicrobial tested was capable of a multiple log_10_-fold reduction of *Pseudomonas* (Fig. 8B, left). With respect to the untreated control (~10^7^ CFU/mL), the lysocin displayed comparable antibacterial activity to that of meropenem and tobramycin (nearly 4-log CFU reduction) and was more effective than cefepime (~2 log CFU reduction). Colistin and levofloxacin were most effective, reducing bacterial viability to or near the LOD (10 CFU/mL).

The amount of endotoxin released by bacteria treated with PyS5-I-GN4 (48 ± 3 EU/mL) was significantly lower than bacteria treated with cefepime (145 ± 11 EU/mL), levofloxacin (285 ± 30 EU/mL), meropenem (157 ± 1 EU/mL), and tobramycin (181 ± 8 EU/mL) (Fig. 8B, right). Colistin-treated bacteria released minimal free endotoxin (2 ± 0 EU/mL), due to the potent anti-endotoxin binding activity of the antibiotic. Endotoxin detected in the untreated control group (126 ± 19 EU/mL), which was significantly higher than the colistin- and lysocin-treated samples, is most likely attributable to cell division events (40). Like PyS2-derived lysocins (23), the collective results from these studies indicate that PyS5-I-GN4 kills *P. aeruginosa* with minimal damage to the outer membrane, thereby allowing endotoxin to remain anchored to the bacterial surface.

### *In vivo* antipseudomonal efficacy of PyS5-I-GN4

Considering that PyS5-I-GN4 is being proposed as a novel alternate therapeutic for treating *P. aeruginosa* infections, a previously described neutropenic murine acute lung infection model of disease (41, 42) was modified to evaluate the *in vivo* antipseudomonal efficacy of the lysocin. In the first set of animal experiments, neutropenic mice were infected intranasally (IN) with 5 × 10^5^ CFU of the nonmucoid *P. aeruginosa* strain PAO1. At 2 h post-infection, mice were treated with a single dose of PyS5-I-GN4 at 25 mg/kg. The lysocin was administered in PBS either intravenously (IV), IN, or both IV and IN (12.5 mg/kg and 12.5 mg/kg). Mice treated IN with PBS only were used as controls. At 24 h post-infection, the lungs, heart, and kidneys from each mouse were removed, homogenized, and plated on *Pseudomonas* Isolation Agar to enumerate the concentration of viable *P. aeruginosa*.

**Fig 9:**
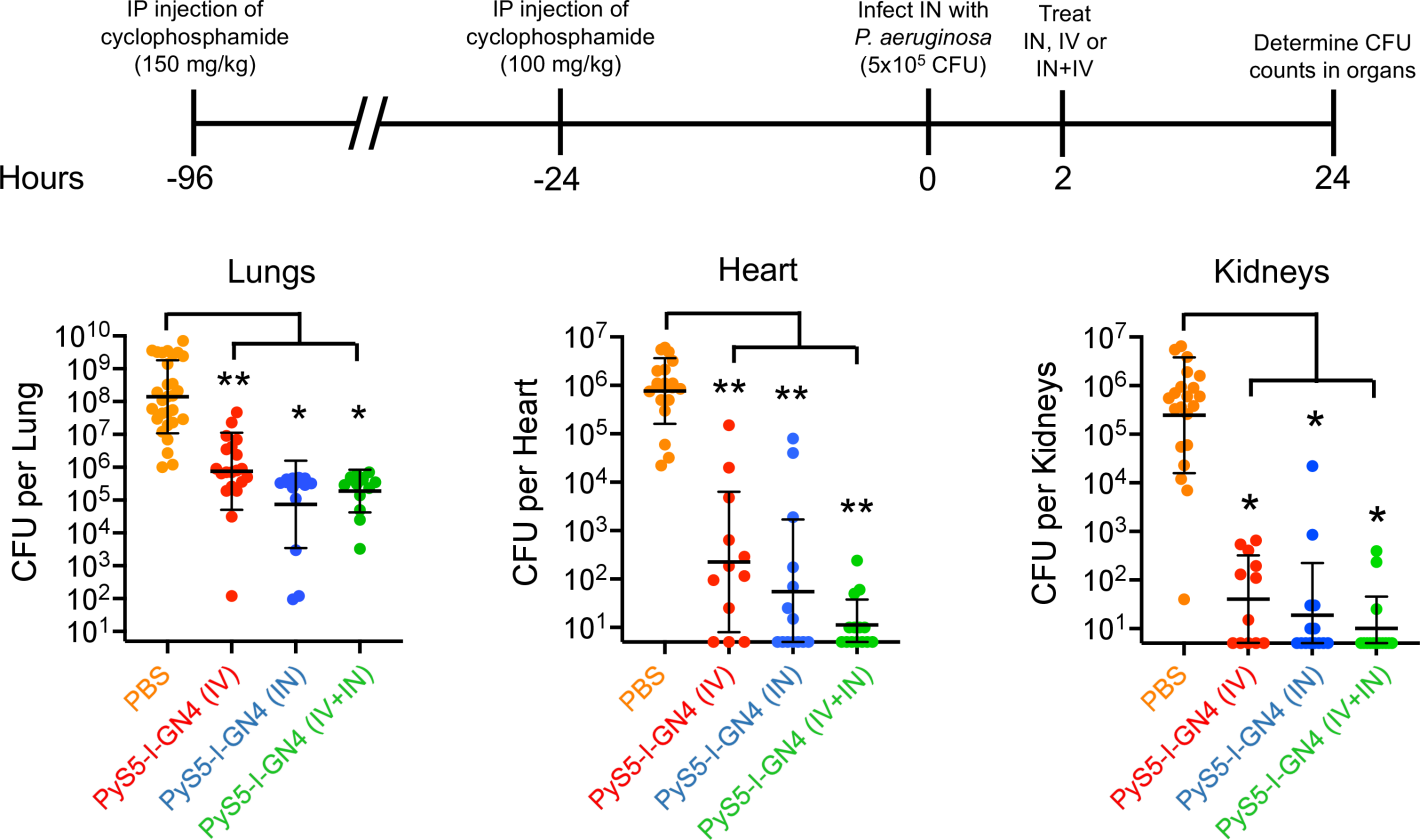
Bacterial clearance from the lungs and secondary organs by PyS5-I-GN4 using an acute neutropenic murine lung infection model. Six- to eight-week-old female C57BL/6 mice were rendered neutropenic by intraperitoneally injecting two doses of cyclophosphamide four days (150 mg/kg) and one day (100 mg/kg) prior to infection. Mice were then infected intranasally (IN) with 5 × 10^5^ CFU of exponential phase *P. aeruginosa* strain PAO1. At 2 h post-infection, mice were treated with PyS5-I-GN4 administered either intravenously (IV) (25 mg/kg; *n* = 20; red), IN (25 mg/kg; *n* = 14; blue), or IN and IV (12.5 mg/kg and 12.5 mg/kg; *n* = 14; green) in PBS. Mice treated IN with PBS only (*n* = 29; orange) were used as untreated controls. At 24 h post-infection, mice were sacrificed and the lungs (left), heart (middle), and kidneys (right) were aseptically removed, homogenized, serially diluted, and plated on *Pseudomonas* Isolation Agar to quantify *P. aeruginosa*. The limit of detection was 5 CFU per organ. Data obtained are from at least two independent experiments. Horizontal lines represent the geometric mean, while error bars signify the geometric standard deviation. Significance levels were determined using an unpaired Student *t*-test. *P < 0.05, **P < 0.01.

Compared to the PBS-treated animals (*n* = 29; 1.4 × 10^8^ CFU per lung), a single dose of lysocin administered either IV (*n* = 20), IN (*n* = 14), or IN plus IV (*n* = 14) decreased bacterial counts in the lungs 2.3, 3.3, and 2.9 logs, respectively (Fig. 9, left). PBS-treated mice were highly bacteremic at 24 h post-infection, with the heart (*n* = 20) and kidneys (*n* = 21) containing 7.6 × 10^5^ and 2.5 × 10^5^ CFU of *Pseudomonas*, respectively. PyS5-I-GN4 administered either IV (*n* = 12), IN (*n* = 14), or IN plus IV (*n* = 14), respectively, lowered *P. aeruginosa* counts in the heart by 3.5, 4.1, and 4.8 logs when compared to the PBS-treated controls (Fig. 9, middle). Similarly, lysocin given either IV (*n* = 12), IN (*n* = 14), or IN plus IV (*n* = 14) reduced pseudomonal viability 3.8, 4.1, and 4.4 logs in the kidneys, respectively (Fig. 9, right). Overall, these results show that PyS5-I-GN4 kills *P. aeruginosa* in the lungs and decreases the bacterial burden in secondary organs after only a single dose.

**Fig 10:**
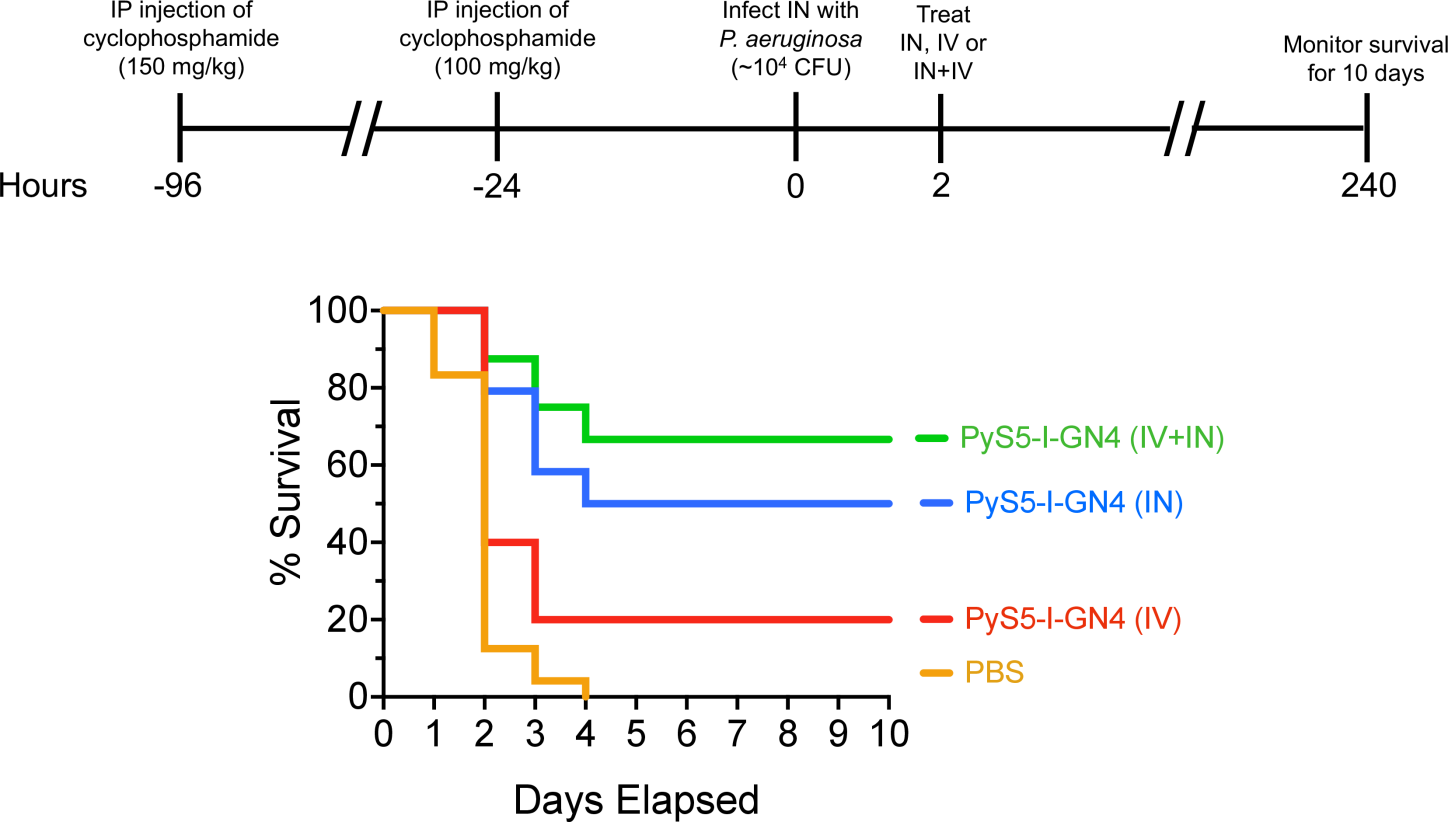
Protection of neutropenic mice from succumbing to *P. aeruginosa* lung infections using a single dose of PyS5-I-GN4. Six- to eight-week-old female C57BL/6 mice were rendered neutropenic by intraperitoneally injecting two doses of cyclophosphamide four days (150 mg/kg) and one day (100 mg/kg) prior to infection. Mice were then infected intranasally (IN) with 5 × 10^3^ to 5 × 10^4^ CFU of exponential phase *P. aeruginosa* strain PAO1. At 2 h post-infection, mice were treated with PBS (*n* = 24; orange) via the IN route or lysocin given either intravenously (IV) (25 mg/kg; *n* = 20; red), IN (25 mg/kg; *n* = 24; blue), or IN and IV (12.5 mg/kg and 12.5 mg/kg; *n* = 24; green) in PBS. Survival was monitored for a total of 10 days. Data obtained are from at least two independent experiments.

In the next series of animal experiments, we evaluated the ability of PyS5-I-GN4 to protect neutropenic mice from succumbing to a pseudomonal lung infection. For these studies, neutropenic mice were infected IN with 5 × 10^3^ to 5 × 10^4^ CFU of *P. aeruginosa* strain PAO1. At 2 h post-infection, mice were treated with a single dose of PyS5-I-GN4 given either IV (25 mg/kg), IN (25 mg/kg), or IN plus IV (12.5 mg/kg and 12.5 mg/kg). Mice treated IN with PBS only served as untreated controls. Mouse survival was then monitored for a total of 10 days. The collective results from these studies showed that approximately 90% of the PBS control group (*n* = 24) died in the first 2 days, with the remaining mice succumbing to the infection by day 4 (Fig. 10). The degree of protection afforded by PyS5-I-GN4 was highly dependent on how the single dose of lysocin was administered. For example, lysocin administered via the IV route (*n* = 20) protected 20% of mice, whereas the survival rate was increased to 50% when PyS5-I-GN4 was given IN (*n* = 24). The highest degree of protection was observed when the lysocin was applied simultaneously both IV and IN. For this group (*n* = 24), the resulting survival rate was nearly 70% at the culmination of the 10-day experiment. Thus, in the absence of neutrophils, a single dose of lysocin is capable of achieving high levels of protection. This finding is attributable to the potent antipseudomonal activity of the therapeutic in the lungs and secondary organs.

## DISCUSSION

In this study, second-generation lysocins were designed and experimentally evaluated in response to the current unmet clinical need for new antipseudomonals (43). One particular lysocin, PyS5-I-GN4, displayed antibacterial activity toward nearly all MDR clinical isolates of *P. aeruginosa* tested and, from a preclinical perspective, exhibited favorable *in vitro* and *in vivo* antipseudomonal properties. The antipseudomonal mechanism of action specific to PyS5-I-GN4 can be assumed based on previous studies involving PyS5 transport across the outer membrane of *P. aeruginosa*, combined with a mechanistic understanding of how lysins (e.g., GN4) and other lysocins (e.g., PyS2-GN4) function (23, 32, 44). In low iron conditions, the gated IROMP, FptA, which is a TonB-dependent transporter, is expressed on the surface of *P*. aeruginosa (e.g., Fig. 1A). When applied externally to *P. aeruginosa* as a purified recombinant protein, the N-terminal disordered region (aa 1–39) of domain I of PyS5-I-GN4 initially binds to FptA (e.g., Fig. 1B). This interaction promotes an allosteric change to FptA that results in the receptor presenting its TonB box motif to the periplasm. The TonB box is then bound by the PMF-linked inner membrane transporter TonB1. In the first PMF-driven event, the TonB1-ExbB-ExbD complex provides the proton gradient required to energize the unfolding of the plug domain of FptA (e.g., Fig. 1C). The unstructured N-terminus of PyS5-I-GN4 can then pass through the newly created channel within FptA to insert its own TonB box (aa 6-14) into the periplasm, which is subsequently bound by another nearby TonB1 (e.g., Fig. 1C). A second PMF-dependent process occurs, in which TonB1, using the energy transduction associated with the PMF, facilitates translocation of PyS5-I-GN4 across the outer membrane (e.g., Fig. 1D). Once in the periplasm (e.g., Fig. 1E), the C-terminal GN4 lysin domain of the lysocin can then cleave the peptidoglycan (e.g., Fig. 1F). The loss in the structural integrity of the peptidoglycan resulting from the cytoplasmic pressure causes a series of events that ultimately kill the bacterial cell, including membrane destabilization, cytoplasmic leakage, and PMF disruption.

Lysocins were strategically designed to deliver a single domain lysin across the outer membrane of *P. aeruginosa*. We assumed that only a limited number of these lysins need to be delivered to the periplasm of *P. aeruginosa* to achieve an observable antibacterial effect, which is based on the structural composition of these enzymes and their relationship to catalytic processivity. Unlike the multimodular lysins derived from phage that infect Gram-positive bacteria, which have an N-terminal catalytic domain linked to a C-terminal CBD, lysins from phage that infect Gram-negative bacteria only comprise a single globular catalytic domain. The CBD of multimodular lysins has been shown to bind to their cell wall-associated carbohydrate with pico- to nanomolar equilibrium affinity constants in order to position the catalytic domain for cell wall cleavage (45, 46); thus, they are largely considered single-use enzymes. For single-domain lysins, the absence of a CBD prevents self-inhibition, enabling the lysin to continuously cleave other bonds within the peptidoglycan structure. Additionally, single domain lysins (15 to 20 kDa) are preferable to multimodular lysins (25 to 40 kDa) when designing lysocins due to their smaller, more compact size (47).

The bactericidal activity of lysocins was improved by removing pyocin domains not directly involved in receptor targeting and translocation (Fig. 2C). This was an unexpected finding, considering that common polysaccharide antigen binding by domain II of the native PyS2 is required for high-efficiency killing of *P. aeruginosa* (29). Interaction with the common polysaccharide antigen increases pyocin concentration on the bacterial surface, consequently allowing the protein to locate its target receptor more efficiently. Compared to the full-length construct, the increased killing kinetics displayed by the truncated lysocin could be simply attributable to its reduced size. It is possible that the truncated lysocin can diffuse more effectively through the LPS layer to subsequently locate its target IROMP receptor. Once bound, the truncated lysocin can also more efficiently translocate through the receptor due to the reduced allosteric hindrance, resulting in a more rapid accumulation in the periplasm. In general, to the best of our knowledge, there are no examples in the literature of an isolated pyocin domain delivering a protein across the outer membrane of *Pseudomonas*. Therefore, for the first time, findings from our collective studies substantiate the use of a single pyocin receptor-binding/translocation domain (e.g., domain I of PyS2, PyS5, and PyG) for targeted drug delivery to *P. aeruginosa*.

Compared to the proof-of-concept lysocin PyS2-GN4 (23) and the truncated PyS2-I-GN4 lysocin, *P. aeruginosa* strain coverage was significantly expanded for PyS5-I-GN4 because of the conservation of the receptor targeted (Tables 1 to 3); however, it remains unclear how the two respiratory strains NR51594 and NR-51603 appear to be insensitive to the lysocin. Results from a multiple sequence alignment comparing FptA sequences from the *P. aeruginosa* strains described in Tables 2 and 3 revealed minimal sequence variation (>99% identity), with no unique amino acid modifications specific to the lysocin-insensitive strains (data not shown). For example, strain NR-51594 (MIC >256 µg/mL) has an identical FptA sequence to that of certain lysocin-sensitive strains, including strain PAO1 (MIC 8 µg/mL). This understanding indicates that the insensitivity to PyS5-I-GN4 displayed by strains NR-51594 and NR-51603 does not appear to be attributable to the amino acid composition of their respective FptA receptors.

For pyocins, the confined space of the IROMP channel requires the protein to partially unfold during translocation. The physicochemical properties of the lysin component could affect the efficiency of outer membrane translocation and/or refolding in the periplasm. Indeed, results from our studies showed that strain coverage of *P. aeruginosa* by PyS5-derived lysocins varied based on the lysin utilized. For example, only 10 of the 16 MDR blood and wound clinical isolates of *P. aeruginosa* tested were sensitive to the PyS5-I-PlyPa204 lysocin (Table S2). Replacing the PlyPa204 lysin with either GN4 (Table 3), PlyPa200, or PlyPa202 (Table S2) resulted in lysocins effective against all 16 clinical isolates. With this in mind, additional studies devoted toward optimizing the lysin component of PyS5-derived lysocins are warranted. By screening an expansive library with highly diverse lysins, it is possible to identify lysocin constructs with more expansive strain coverage than that of PyS5-I-GN4.

Studies attempting to promote *P. aeruginosa* to acquire resistance to lysocins have not been performed to date. However, it is hypothesized that evolving resistance to lysocins will be challenging for the targeted bacteria due to the likely detrimental ramifications associated with resistance mechanisms that disrupt iron acquisition (pyocin component) or alter the chemical composition of the peptidoglycan (lysin component) (48–55). Furthermore, due to their high specificity for the IROMPs targeted on the bacterial surface, lysocins are narrow-spectrum therapeutics (Table S1) that avoid collateral damage to the microflora, thereby reducing the evolutionary pressure on non-pseudomonal bacterial species to develop resistance.

Serum inactivation observed for most single-domain lysins limits their therapeutic use for treating Gram-negative bacteria to non-systemic applications (22). Alternatively, an important property conserved among lysocins is their ability to retain antibacterial activity in the presence of complex matrices, including serum (Fig. 6C) (23). Importantly, the bactericidal potency of PyS5-I-GN4 was comparable in both unheated (Fig. 6C, red) and heat-inactivated human serum (Fig. 6C, black). The ability of lysocins to rapidly kill *P. aeruginosa* in serum independent of a functional complement system further highlights their therapeutic potential for use in immunocompromised individuals. In general, this particular engineering methodology affords lysins the flexibility of potentially being used clinically as therapeutics that comprehensively cover both superficial and systemic infections caused by Gram-negative bacterial pathogens.

Administering lysocins formulated as a monotherapy for treating chronic lung infections in CF patients could prove challenging. The micromolar concentration of iron observed in respiratory secretions and sputum from patients with CF may be sufficient to reduce the expression of IROMPs targeted by lysocins for outer membrane translocation. However, this can potentially be circumvented by supplementing the lysocin with natural (e.g., lactoferrin and deferoxamine) or synthetic (e.g., deferasirox) FDA-approved iron chelators (56). Iron chelators alone can inhibit biofilm formation in CF sputum isolates of *P. aeruginosa* (57). Notably, the combination of tobramycin and iron chelators was synergistic in reducing both the biomass of and bacterial viability within established pseudomonal biofilms (58). Considering that the broth microdilution assays and most of the *in vitro* killing assays described in this study were performed in the presence of an iron chelator (ethylenediamine-N,N’-bis(2-hydroxyphenylacetic acid; EDDHA), we are confident that lysocins will retain their bactericidal properties when combined with the aforementioned FDA-approved iron chelators. Furthermore, we have demonstrated that lysocins are effective in treating infections *in vivo* without the need for additional buffer supplementation (Fig. 9 and 10, and [23]).

In a neutropenic background, the *in vivo* antipseudomonal efficacy of PyS5-I-GN4 was established using an acute murine lung infection model of disease. A single dose of the lysocin both reduced *P. aeruginosa* viability multiple log_10_-fold in the lungs and secondary organs of neutropenic mice (Fig. 9) and protected nearly 70% of the neutropenic mice from succumbing to the lung infection (Fig. 10). The degree of protection provided by the lysocin was highly dependent on the route used to administer the drug. In addition to optimizing how the lysocin is administered, the antipseudomonal efficacy of PyS5-I-GN4 could be further improved by implementing a multi-dose regimen. Moreover, through the aid of an uncompromised immune system, the therapeutic effects of the lysocin would expectedly be amplified in a non-neutropenic background.

Although the *in vivo* antibacterial efficacy of PyS5-I-GN4 was validated for specifically treating pseudomonal lung infections, it is assumed that its therapeutic use could be expanded to treating bloodstream and burn wound infections caused by *P. aeruginosa*. This conclusion was formulated based on (i) the validated *in vivo* antibacterial activity of other lysocins for treating bloodstream infections, (ii) the *in vitro* bactericidal activity exhibited by PyS5-I-GN4 in the presence of human serum (Fig. 6C), and (iii) the inherent susceptibility of *P. aeruginosa* to lysocin in burn wounds and the bloodstream due to the upregulation of IROMPs caused by iron deprivation (23, 59–63).

In summary, we successfully engineered second-generation lysocins that deliver functional lysins to their peptidoglycan substrate in most *P. aeruginosa* strains tested. Due to the conservation of the receptor targeted (FptA), the PyS5-I-GN4 lysocin (Fig. 3A) exhibited highly specific antipseudomonal activity (Table S1) against nearly all (95%) of the clinically relevant MDR strains of *P. aeruginosa* tested (Tables 2 and 3). This indicates that a functional lysin can be delivered through FptA. Additional *in vitro* highlights for PyS5-I-GN4 include bactericidal activity at low concentrations (Fig. 4), rapid bacterial killing kinetics in lung surfactant and serum (Fig. 6B and C), antibiofilm properties (Fig. 7), an absence of cytotoxicity toward human cells (Fig. 8A), and decreased endotoxin release compared to standard-of-care antibiotics (Fig. 8B). Administering a single dose of lysocin to neutropenic mice with an acute *P. aeruginosa* lung infection reduced the bacterial burden in the lung and secondary organs (Fig. 9) and consequently was highly protective (Fig. 10). Notably, in addition to the GN4 lysin, the application of PyS5-derived lysocins can be broadly expanded to incorporate other lysin candidates (e.g., Fig. 5). The collective findings from our studies indicate that second-generation lysocins, such as PyS5-I-GN4, have the potential to serve as novel antipseudomonals for treating both superficial and systemic infections caused by *P. aeruginosa*, particularly those that are MDR.

## MATERIALS AND METHODS

### Bacterial strains and culture conditions

Bacterial strains used in this study are outlined in Table S3 and were stored at −80°C. Unless stated otherwise, bacterial strains were routinely grown aerobically overnight in either Brain Heart Infusion (BHI, Gram-positive bacteria; BD Biosciences) or CAA medium (Gram-negative bacteria; 5 g/L Casamino acids, 5.2 mM K_2_HPO_4_, 1 mM MgSO_4_; Fisher Scientific). The NR-51515 to NR-51614 MDR respiratory, blood, and wound clinical isolates of *P. aeruginosa* tested in Tables 2 and 3 and Table S2 is from the Multidrug-Resistant Organism Repository and Surveillance Network (MRSN) *Pseudomonas aeruginosa* Diversity Panel at the Walter Reed Army Institute for Research. This panel, which was collected from all over the world over a span of 11 years, comprises genetically diverse clinical isolates of *P. aeruginosa* that maximizes phylogenetic distance and pan-genome diversity (64).

### Molecular cloning

Genes encoding the translated pyocin candidates PyS2 (GenBank NP_249841), PyS5 (GenBank WP_003115311) and PyG (GenBank WP_170835785), as well as the lysin candidates GN3 (GenBank WP_012273008), GN4 (GenBank YP_002284361), PlyPa03 (GenBank WP_070344501), PlyPa103 (GenBank AQZ96894), PlyPa200 (GenBank WP_120123400), PlyPa202 (GenBank WP_108546479), PlyPa204 (GenBank WP_197853647), and T4L (GenBank NP_049736), were synthesized and codon-optimized for protein expression in *Escherichia coli* (GeneWiz, Inc.). The *gn4* and *pys2-gn4* genes were cloned into the *E. coli* expression vector pET28a as previously mentioned (23). The template DNA and oligonucleotide primers used to generate the polymerase chain reaction (PCR) products necessary for designing each lysocin construct described in this study are outlined in Table S4. Additionally, the nucleotide sequence of each primer is described in Table S5. Each lysocin construct was created using the NEBuilder HiFi DNA Assembly Cloning Kit (New England Biolabs). The pyocin and lysin gene fragments were initially amplified using PCR. The standard 50 µL PCR reaction consisted of 1 ng template DNA, 1× Q5 Reaction Buffer, 0.2 mM dNTPs, 0.5 µM of each oligonucleotide primer, and 1 U of Q5 DNA polymerase (New England Biolabs). The thermocycler heating conditions were 98°C for 30 s, 35 cycles of 98°C for 10 s, 60°C for 30 s, 72°C for 30 s per kb, followed by 72°C for 2 min. Next, a 20 µL reaction consisting of 1× NEBuilder HiFi DNA Assembly Master Mix, the PCR product(s), and linearized pET28a digested with NcoI and BamHI restriction endonucleases (New England Biolabs) was incubated at 50°C for 15 min. Finally, 2 µL from each assembly reaction was transformed into chemically competent *E. coli* DH5α.

### Protein expression and purification

GN4 and PyS2-GN4 were expressed and purified using previously described methods (23). Using the expression strain *E. coli* BL21(DE3), each additional lysocin construct was expressed at either 18°C for 16–18 h (PyG-I-GN4 and all PyS5-derived lysocins except PyS5-I-GN4) or 37°C for 4 h (PyS2-I-GN4 and PyS5-I-GN4) in Luria-Bertani medium (BD Biosciences) supplemented with 50 µg/mL kanamycin (Fisher Scientific). Protein expression was induced at mid-log phase (OD_600nm_ = 0.5) with 1 mM isopropyl β-D-1-thiogalactopyranoside (Biosynth). The bacteria were then harvested, washed, and resuspended to a cell density of 100 mg/mL in 50 mM Tris-HCl, pH 7.5, 200 mM NaCl, and 1 mM phenylmethylsulfonyl fluoride (Sigma-Aldrich). The cells were lysed on ice with a Q125 Sonicator (Qsonica) for 10 min using 10 s on/off cycles with 50% amplitude. The lysate was cleared by centrifugation at 13,000 RPM for 1 h at 4°C. The soluble lysate fraction was dialyzed against 10 mM sodium phosphate, pH 7.0, and then subsequently sterile filtered with a 0.2 µm syringe filter to generate the crude lysate.

Using an AKTA fast protein liquid chromatography system (GE Healthcare Life Sciences), the crude lysate was applied to either a 5 mL HiTrap CM FF (all lysocins except PyG-I-GN4; Cytiva) or HiTrap SP HP cation exchange column (PyG-I-GN4; Cytiva) in 10 mM sodium phosphate, pH 7.0 (Fisher Scientific). Protein was eluted from the column using a 20 column volume linear salt gradient from 0 to 250 mM NaCl (Fisher Scientific). Elution fractions comprising the lysocin were combined and dialyzed against 50 mM Tris-HCl, pH 7.5, 200 mM NaCl. The dialyzed protein sample was concentrated using an Amicon Ultra Ultracel-10K spin filter (EMD Millipore). The concentrated protein sample was finally applied to a HiLoad 16/60 Superdex 200 Prep Grade column (Cytiva) in 50 mM Tris-HCl, pH 7.5, 200 mM NaCl. Elution fractions comprising highly pure lysocin were combined, concentrated, supplemented with 10% (vol/vol) glycerol, and sterile filtered. Aliquots were stored at −80°C until further needed.

### Time-kill assay

Using a 96-well untreated microtiter plate, *P. aeruginosa* strain 453 (PyS2-derived lysocins) or PAO1 (PyS5-derived lysocins) at an initial concentration of 10^6^ CFU/mL were statically treated with lysocin at 0.5 µM in either CAA medium supplemented with 0.5 mg/mL EDDHA (Complete Green Company), human serum (pooled human male AB plasma; Sigma-Aldrich), heat-inactivated human serum (pooled human male AB plasma; Sigma-Aldrich) or 50% (vol/vol) beractant (Survanta; Abbvie) for up to 6 h at 37°C. For the studies using beractant, 20 mM sodium phosphate, pH 7.0, was used as the diluent. Bacteria without lysocin treatment served as untreated controls. At various time intervals, an aliquot was removed from each sample, serially diluted in buffer or media corresponding to the same treatment conditions, and plated on CAA agar to quantitate the CFU/mL concentration of viable bacteria. The LOD was 10 CFU/ml. Error bars correspond to the ± standard error of the mean (SEM) of triplicate biological replicates.

### 4-hour killing assay

Using a 96-well untreated microtiter plate, *P. aeruginosa* strain 453 (PyS2-derived lysocins) or PAO1 (PyS5- and PyG-derived lysocins) at an initial concentration of 10^6^ CFU/mL were statically treated with lysocin at 0.5 µM in CAA medium supplemented with 0.5 mg/mL EDDHA for 4 h at 37°C. PyS5-I-GN4 and PyG-I-GN4 were tested at 0.5 to 512 µg/mL (~10 nM to 13 µM) for the dose-response killing assays. *P*-values for the dose-response experiments were calculated using an unpaired Student *t*-test. ns, not significant, ****P < 0.0001. Antimicrobial concentrations at 5× MIC were used for the assays comparing PyS5-I-GN4 and various standard-of-care antipseudomonal antibiotics. For the pH studies, *P. aeruginosa* were treated with PyS5-I-GN4 in 50 mM sodium phosphate buffer at pH 6.0 to 8.0. For the salt experiments, *P. aeruginosa* were treated with PyS5-I-GN4 in 20 mM sodium phosphate, pH 7.3, supplemented with 0 to 250 mM NaCl. *P*-values for the pH- and salt-based killing assays were calculated using a one-way ANOVA Brown-Forsythe test. ns, not significant. Untreated bacteria served as a negative control for antipseudomonal activity for each assay. All samples were serially diluted in buffer or media corresponding to the same treatment conditions and then plated on CAA agar to quantitate the CFU/mL concentration of viable bacteria. The LOD was 10 CFU/mL. Error bars correspond to the ± SEM of triplicate biological replicates.

### Muralytic assay

An overnight culture of *P. aeruginosa* strain 453 was diluted 1:100 in BHI medium and grown for 16 to 18 h at 37°C with aeration. The bacteria were harvested, washed, and diluted 3.2-fold (with respect to the initial culture volume) in 50 mM Tris-HCl, pH 7.5, supplemented with 0.75% (wt/vol) Bacto agar (BD Biosciences). After the mixture was autoclaved, the muralytic activity of each lysocin was assessed by spotting 25 pmol of purified protein on the autoclaved *P. aeruginosa*. Buffer was spotted as a negative control for muralytic activity. Clearing zones observed after 20–24 h at 37°C correspond to peptidoglycan cleavage.

### Modified broth microdilution assay

The MIC of the various antimicrobials tested was determined using a modified version of the broth microdilution assay previously described by the Clinical and Laboratory Standards Institute (65). Briefly, using an untreated 96-well round bottom microtiter plate, bacteria at ~ 2.5 to 5 × 10^5^ CFU/mL were incubated statically in triplicate with antimicrobial concentrations from 0.002 up to 256 µg/mL. For the lysocins assayed, these antimicrobial concentrations equate to molar concentrations of approximately 50 pM to 7 µM. Each microdilution assay was performed in either Mueller Hinton Broth (for Gram-positive bacteria; BD Biosciences) or CAA medium (for Gram-negative bacteria) supplemented with 0.5 mg/mL EDDHA for approximately 20 h at 37°C with humidity. Due to the iron-depleted growth conditions, some bacterial strains required an additional 24 h for observable growth. Growth (bacteria absent antimicrobial) and sterility controls (growth medium only) were included for each plate assayed. The MIC was defined as the lowest antimicrobial concentration that inhibits observable bacterial growth, which was equated to pellet formation at the bottom of the respective well.

### Antibiofilm activity

*P. aeruginosa* strain PAO1 at ~ 10^6^ CFU/mL in tryptic soy broth (BD Biosciences) with 0.2% (wt/vol) glucose (Fisher Scientific) was aliquoted at 100 µL into individual wells of a MBEC Assay Biofilm Inoculator 96-well plate (Innovotech). Sterility controls, consisting of growth medium only, were included. Biofilms were grown on the plastic pegs attached to the MBEC plate lid for 24 h at 37°C with shaking at 110 RPM. The peg lid containing mature biofilms was washed twice with 20 mM sodium phosphate, pH 7.0, air-dried, and then transferred to a new 96-well flat-bottomed microtiter plate containing 0.5 to 256 µg/mL (13 nM to 7 µM) of PyS5-I-GN4 in 150 µL CAA medium with 0.2% (wt/vol) glucose and 0.5 mg/mL EDDHA. Growth controls, consisting of biofilms grown in the absence of lysocin treatment, were included. The biofilms were treated for 24 h at 37°C under static conditions. The biofilms were again washed twice with buffer and air-dried.

For the biofilm disruption assays, the peg lid was transferred to a 96-well flat-bottomed microtiter plate containing 175 µL 0.05% (wt/vol) crystal violet (Sigma-Aldrich) per well. After 10 min at room temperature, the peg lid was washed twice with buffer, air-dried, and the residual crystal violet bound to the peg was eluted in 200 µL 33% (vol/vol) acetic acid (Fisher Scientific) per well. The contents of each well were mixed, and the absorbance at an OD_600nm_ was measured using a SpectraMax M5 microplate reader (Molecular Devices). The data were normalized to the absorbance values of the growth controls.

In parallel, bacterial viability within the lysocin-treated *P. aeruginosa* biofilms was measured using a previously described method (66). Briefly, a duplicate MBEC plate lid containing mature *P. aeruginosa* biofilms treated with or without lysocin was transferred to a 96-well microtiter plate containing 200 µL buffer per well. The plate was sonicated in a water bath for 30 min at room temperature, with the contents of each well subsequently serially diluted in buffer and plated on CAA agar. The LOD was 10 CFU/mL. Error bars correspond to ± SEM of triplicate biological replicates. *P*-values were calculated using an unpaired Student *t*-test. ns, not significant, *P < 0.05, **P < 0.01, ***P < 0.001.

### Synergy between lysocin and antipseudomonal antibiotics

A checkerboard analysis was performed to evaluate antibacterial potency when combining PyS5-I-GN4 with a collection of antipseudomonal antibiotics, compared to their individual activities. Antibiotics tested include cefepime, colistin sulfate, levofloxacin, meropenem, piperacillin-tazobactam, and tobramycin (Fisher Scientific). Using an untreated 96-well round-bottom plate, antibiotic concentrations from 0.002 to 2× MIC were mixed with PyS5-I-GN4 concentrations from 0.03 to 2× MIC in a total volume of 100 µL CAA medium supplemented with 0.5 mg/mL EDDHA. Next, 10 µL of *P. aeruginosa* strain PAO1 were added to each well to obtain an initial concentration of ~ 2.5 to 5 × 10^5^ CFU/mL. After a static incubation for approximately 20 h at 37°C, bacterial growth was assessed. The FICI for each antimicrobial combination was calculated using the following formula: A/MIC_A_ + B/MIC_B_ = FIC_A_ + FI_CB_ = FICI, where *A* and *B* are the MIC of each antimicrobial in combination (in a single well) and *MIC*_*A*_ and *MIC*_*B*_ are the MIC of each antimicrobial individually. FICI values were interpreted as synergistic if the FICI was ≤0.5, additive if the FICI was >0.5 and ≤1, indifferent if the FICI was >1 and ≤4, and antagonistic if the FICI value was >4 (67).

### Measuring cytotoxicity toward human red blood cells

A previously established method (68) was used to evaluate the hemolytic properties of PyS5-I-GN4. Briefly, human blood from healthy adult donors was collected in an EDTA-containing conical tube at the Rockefeller University Hospital. This study was approved by our Institutional Review Board, and all adult subjects provided written informed consent. The red blood cells were pelleted using low-speed centrifugation, washed three times, and resuspended to a 10% (vol/vol) concentration in PBS, pH 7.4 (Fisher Scientific). In a 96-well microtiter plate, the red blood cell solution was mixed 1:1 with PyS5-I-GN4 at final concentrations from 0.5 to 256 μg/mL. Red blood cells mixed with buffer or 0.1% (vol/vol) Triton X-100 (Fisher Scientific) represented negative and positive controls for hemolytic activity, respectively. Following a 4-hour incubation at 37°C with 5% CO_2_, intact cells were pelleted using low-speed centrifugation. The supernatant from each sample was transferred to a new microtiter plate, and the absorbance was measured spectrophotometrically at an OD_405nm_ using a SpectraMax M5 microplate reader. All error bars correspond to the ± SEM of triplicate biological replicates. *P*-values were calculated using a one-way ANOVA Brown-Forsythe test. ns, not significant, ****P < 0.0001.

### Endotoxin release

A previously described method (23) was used to measure the concentration of endotoxin released from *P. aeruginosa* following treatment with various antimicrobials. To summarize, *P. aeruginosa* strain PAO1 at an initial concentration of 10^6^ CFU/mL was treated statically with 5× MIC concentrations of either PyS5-I-GN4, colistin, cefepime, levofloxacin, meropenem, or tobramycin for 4 h at 37°C in CAA medium. Growth medium alone was used as a blank, while bacteria in growth medium without antimicrobial served as untreated controls. An aliquot was removed, serially diluted in growth medium, and plated on CAA agar to quantify the CFU/mL concentration of surviving bacteria. The remaining volume from each sample was centrifuged at 2,000 × *g* for 10 min to remove intact cells and large cellular debris. The supernatant was then passed through a 0.2 µm syringe filter. Endotoxin concentration (EU/mL) in the filtered supernatant was quantitated using a ToxinSensor Chromogenic LAL Endotoxin Assay Kit (GenScript). Error bars correspond to the ± SEM of duplicate biological replicates. *P*-values were calculated using an unpaired Student *t*-test. *P < 0.05, **P < 0.01, ***P < 0.001.

### Neutropenic murine model of acute lung infection

All research protocols were approved by the Rockefeller University Institutional Animal Care and Use Committee. A previously described acute murine lung infection model of disease (41, 42) was adapted to elucidate the *in vivo* antipseudomonal efficacy of PyS5-I-GN4. Six- to eight-week-old female C57BL/6 mice were rendered neutropenic by intraperitoneally injecting two doses of cyclophosphamide four days (150 mg/kg) and one day (100 mg/kg) prior to infection. Mice were then infected IN with 5 × 10^5^ CFU of exponential phase *P. aeruginosa* strain PAO1. At 2 h post-infection, mice were treated with PyS5-I-GN4 administered either IV (25 mg/kg), IN (25 mg/kg), or IN and IV (12.5 mg/kg and 12.5 mg/kg) in PBS. Mice treated IN with PBS only were used as untreated controls. At 24 h post-infection, mice were sacrificed, and the lungs, heart, and kidneys were aseptically removed. The organs were homogenized in 0.5 mL PBS using a Stomacher 80 Biomaster homogenizer (Seward). The homogenized samples were serially diluted in buffer and plated on *Pseudomonas* Isolation Agar (Hardy Diagnostics) to quantify surviving *P. aeruginosa*. The limit of detection was 5 CFU per organ. *P*-values were calculated using an unpaired Student *t*-test. *P < 0.05, **P < 0.01. For the survival experiments, neutropenic mice were infected IN with between 5 × 10^3^ to 5 × 10^4^ CFU of exponential phase *P. aeruginosa* strain PAO1. At 2 h post-infection, mice were treated with PyS5-I-GN4 given either IV (25 mg/kg), IN (25 mg/kg), or IN and IV (12.5 mg/kg and 12.5 mg/kg) in PBS. Untreated controls consisted of mice treated IN with PBS only. Survival was monitored for 10 days. All data obtained are from at least two independent experiments.

## SUPPLEMENTAL MATERIAL

Supplemental tables aac.01312-25-s0001.docx Tables S1 to S5.

ASM does not own the copyrights to Supplemental Material that may be linked to, or accessed through, an article. The authors have granted ASM a non-exclusive, world-wide license to publish the Supplemental Material files. Please contact the corresponding author directly for reuse.
