# Supplementary material for: Second-generation lysocins as therapeutics for treating Pseudomonas aeruginosa infections
Source: Antimicrob Agents Chemother. 2025 Nov 12;69(12):e01312-25. doi: 10.1128/aac.01312-25 (PMC12691632; doi:10.1128/aac.01312-25)
Supplement: Supplemental tables — Tables S1 to S5. [file aac.01312-25-s0001.docx]

**Supplemental Material**

**Table S1.** Antibacterial activity range of PyS5-I-GN4.

| Bacterial species | Strain | MIC (μg/mL) |
| --- | --- | --- |
| *P. aeruginosa* | PAO1 | 4 |
| *Acinetobacter baumannii* | ATCC BAA-1792 | >256 |
| *Bacillus anthracis* | ∆Sterne | >256 |
| *Citrobacter freundii* | ATCC 8090 | >256 |
| *Enterobacter cloacae* | NR-50391 | >256 |
| *Enterococcus faecalis* | V12 | >256 |
| *Enterococcus faecium* | EFSK2 | >256 |
| *Escherichia coli* | L22-1 | >256 |
| *Klebsiella pneumoniae* | ATCC 700603 | >256 |
| *Listeria monocytogenes* | HER1184 | >256 |
| *Pseudomonas* spp. | HPB0071 | >256 |
| *Pseudomonas entomophila* | AR455 | >256 |
| *Pseudomonas luteola* | AR479 | >256 |
| *Pseudomonas oryzihabitans* | AR456 | >256 |
| *Pseudomonas putida* | AR478 | >256 |
| *Salmonella* spp. Group D | AR396 | >256 |
| *Staphylococcus aureus* | NRS382 | >256 |
| *Streptococcus agalactiae* | 090R | >256 |

**Table S2.** MIC analysis of PyS5-I-PlyPa200, PyS5-I-PlyPa202 and PyG-I-PlyPa204 towards MDR blood and wound clinical isolates of *P. aeruginosa*.

|  | MIC (μg/mL) | | |
| --- | --- | --- | --- |
| *P. aeruginosa* Strain | PyS5-I-PlyPa200 | PyS5-I-PlyPa202 | PyS5-I-PlyPa204 |
|  |  |  |  |
| PAO1 | 8 | 8 | 16 |
| NR-51516 | 4 | 8 | 8 |
| NR-51517 | 8 | 8 | 32 |
| NR-51525 | 8 | 16 | >128 |
| NR-51530 | 128 | 128 | >128 |
| NR-51536 | 32 | 32 | 32 |
| NR-51546 | 32 | 64 | >128 |
| NR-51549 | 8 | 32 | 32 |
| NR-51556 | 4 | 8 | 16 |
| NR-51557 | 8 | 8 | >128 |
| NR-51565 | 8 | 8 | 32 |
| NR-51568 | 8 | 8 | 16 |
| NR-51569 | 8 | 16 | >128 |
| NR-51574 | 8 | 8 | 16 |
| NR-51579 | 16 | 16 | >128 |
| NR-51593 | 8 | 16 | 32 |

**Table S3.** Bacterial strains used in this study.

| Species | Strain | Source | Notes |
| --- | --- | --- | --- |
| *Acinetobacter baumannii* | ATCC BAA-1792 | ATCC | MDR, CRAB |
| *Bacillus anthracis* | ∆Sterne | RUBC | pXO1^-^, pXO2^-^ |
| *Citrobacter freundii* | ATCC 8090 | ATCC |  |
| *Enterobacter cloacae* | NR-50391 | BEI Resources, NIAID |  |
| *Enterococcus faecalis* | V12 | RUBC | VSE |
| *Enterococcus faecium* | EFSK2 | Alexander Tomasz | VRE |
| *Escherichia coli* | L22-1 | (1) | ESBL-producing |
| *Klebsiella pneumoniae* | ATCC 700603 | ATCC | MDR, ESBL-producing |
| *Listeria monocytogenes* | HER1184 | RUBC |  |
| *Pseudomonas aeruginosa* | 443-453 | NYP/WCMC |  |
| *Pseudomonas aeruginosa* | AR465-AR474 | NYU |  |
| *Pseudomonas aeruginosa* | NR-51515-51614 | BEI Resources, NIAID | MRSN Diversity Panel, MDR |
| *Pseudomonas aeruginosa* | PAO1 | ATCC |  |
| *Pseudomonas aeruginosa* | RH8-RH14 | CU | CFI, MDR, mucoid |
| *Pseudomonas aeruginosa* | RH22, RH24 | CU | CFI, MDR, nonmucoid |
| *Pseudomonas* spp. | HPB0071 | BEI Resources, NIAID |  |
| *Pseudomonas entomophila* | AR455 | RUBC |  |
| *Pseudomonas luteola* | AR479 | RUBC |  |
| *Pseudomonas oryzihabitans* | AR456 | RUBC |  |
| *Pseudomonas putida* | AR478 | RUBC |  |
| *Salmonella* spp. Group D | AR396 | RUBC |  |
| *Staphylococcus aureus* | NRS382 | NARSA Collection | MRSA |
| *Streptococcus agalactiae* | 090R | RULC |  |

ATCC, American Type Culture Collection; CFI, Cystic Fibrosis Isolate; CRAB, Carbapenem-Resistant *A. baumannii*; CU, Columbia University Irving Medical Center; ESBL, Extended Spectrum β-Lactamase; MDR, Multidrug-Resistant; MRSA, Methicillin-Resistant *S. aureus*; MRSN, Multidrug-Resistant Organism Repository and Surveillance Network; NARSA, Network on Antimicrobial Resistance in *S. aureus*; NIAID, National Institute of Allergies and Infectious Diseases; NYP/WCMC, NewYork Presbyterian/Weill Cornell Medical Center; NYU, New York University Langone Medical Center; RUBC, The Rockefeller University Bacterial Collection; RULC, The Rockefeller University Lancefield Collection; VRE, Vancomycin-Resistant *Enterococcus*; VSE, Vancomycin-Sensitive *Enterococcus*

**Table S4.** Outline of components required for PCR and molecular cloning.

| Construct Name | PCR Product | Template | Oligonucleotides |
| --- | --- | --- | --- |
| PyS2-I-GN4 | *pyS2-I* | *pyS2* | PyS2 F and PyS2-I R |
|  | *gn4* | *gn4* | GN4 F and GN4 R |
| PyS5-I-GN4 | *pyS5-I* | *pyS5* | PyS5 F and PyS5-I R |
|  | *gn4* | *gn4* | GN4 F and GN4 R |
| PyG-I-GN4 | *pyG-I* | *pyG* | PyG F and PyG-I R |
|  | *gn4* | *gn4* | GN4 F and GN4 R |
| PyS5-I-T4L | *pyS5-I* | *pyS5* | PyS5 F and PyS5-I R |
|  | *t4l* | *t4l* | T4L F and T4L R |
| PyS5-I-GN3 | *pyS5-I* | *pyS5* | PyS5 F and PyS5-I R |
|  | *gn3* | *gn3* | GN3 F and GN3 R |
| PyS5-I-PlyPa03 | *pyS5-I* | *pyS5* | PyS5 F and PyS5-I R |
|  | *plypa03* | *plypa03* | PlyPa03 F and PlyPa03 R |
| PyS5-I-PlyPa103 | *pyS5-I* | *pyS5* | PyS5 F and PyS5-I R |
|  | *plypa103* | *plypa103* | PlyPa103 F and PlyPa103 R |
| PyS5-I-PlyPa200 | *pyS5-I* | *pyS5* | PyS5 F and PyS5-I R |
|  | *plypa200* | *plypa200* | PlyPa200 F and PlyPa200 R |
| PyS5-I-PlyPa202 | *pyS5-I* | *pyS5* | PyS5 F and PyS5-I R |
|  | *plypa202* | *plypa202* | PlyPa202 F and PlyPa202 R |
| PyS5-I-PlyPa204 | *pyS5-I* | *pyS5* | PyS5 F and PyS5-I R |
|  | *plypa204* | *plypa204* | PlyPa202 F and PlyPa204 R |

**Table S5.** Sequences of oligonucleotide primers used in this study.

| Oligonucleotide | Nucleotide Sequence |
| --- | --- |
| PyS2 F | 5’-aactttaagaaggagatataCCATGGCCGTGAACGATTATG-3’ |
| PyS2-I R | 5’-gctaccgctgccgctaccTTTGTAGTCTGCCTCAAC-3’ |
| GN4 F | 5’-ggtagcggcagcggtagcCGCACCAGCCAGCGCG -3’ |
| GN4 R | 5’-gtcgacggagctcgaattcggatccTTAGCTCAGCGGTTCCAGAAACA  GTGC-3’ |
| T4L F | 5’-ggtagcggcagcggtagcAACATCTTCGAAATGCTGCGC-3’ |
| T4L R | 5’-gtcgacggagctcgaattcggatccTTACAGATTTTTATAGGCATCC-3’ |
| GN3 F | 5’-ggtagcggcagcggtagcCGCACCAGCCAGCGTGGCC-3’ |
| GN3 R | 5’-gtcgacggagctcgaattcggatccTTAGGCGGCACCCAGAAACAG-3’ |
| PlyPa03 F | 5’-ggtagcggcagcggtagccgtacatcccaacgaggc-3’ |
| PlyPa03 R | 5’-gtcgacggagctcgaattcggatccTCACGATAGCGGCTCCAG-3’ |
| PyS5 F | 5’-aactttaagaaggagatataATGTCCAATGACAACGAAG-3’ |
| PyS5-I R | 5’-tgccgctaccTAGCGCTTTTTGCTTGTTG-3’ |
| PyG F | 5’-aactttaagaaggagatataATGGCCCGCCCGATCGCG-3’ |
| PyG-I R | 5’-tgccgctaccCGCCGGCATGGCGTACGTG-3’ |
| PlyPa103 F | 5’-ggtagcggcagcggtagcgcgtggtccgctaaagtgag-3’ |
| PlyPa103 R | 5’-gtcgacggagctcgaattcggatccTTACGCTGCTGCACGGCG-3’ |
| PlyPa200 F | 5’-ggtagcggcagcggtagcCGCACGAGTCAGAAAGGC-3’ |
| PlyPa200 R | 5’-CGGAGCTCGAATTCGGATCCTTACGCTTTGCTCAGAAACAGC-3’ |
| PlyPa202 F | 5’-ggtagcggcagcggtagcCGCACGAGTCAGCGCGGCC-3’ |
| PlyPa202 R | 5’-CGGAGCTCGAATTCGGATCCTTACGCCGCTTCCAGAAACAG-3’ |
| PlyPa204 R | 5’-CGGAGCTCGAATTCGGATCCTTACACCGCTTCCAGAAACAGC-3’ |

**References**

1. Satlin MJ, Chavda KD, Baker TM, Chen L, Shashkina E, Soave R, Small CB, Jacobs SE, Shore TB, van Besien K, Westblade LF, Schuetz AN, Fowler VG, Jr., Jenkins SG, Walsh TJ, Kreiswirth BN. 2018. Colonization With Levofloxacin-resistant Extended-spectrum beta-Lactamase-producing Enterobacteriaceae and Risk of Bacteremia in Hematopoietic Stem Cell Transplant Recipients. Clin Infect Dis 67:1720-1728.
